# Self-assembly nanovaccine containing TLR7/8 agonist and STAT3 inhibitor enhances tumor immunotherapy by augmenting tumor-specific immune response

**DOI:** 10.1136/jitc-2021-003132

**Published:** 2021-08-26

**Authors:** Lele Zhang, Jiacheng Huang, Xiaona Chen, Caixu Pan, Yong He, Rong Su, Danjing Guo, Shengyong Yin, Shuai Wang, Lin Zhou, Jianxiang Chen, Shusen Zheng, Yiting Qiao

**Affiliations:** 1 Division of Hepatobiliary and Pancreatic Surgery, Department of Surgery, The First Affiliated Hospital, Zhejiang University School of Medicine, Hangzhou, People's Republic of China; 2 NHC Key Laboratory of Combined Multi-organ Transplantation, Hangzhou, People's Republic of China; 3 College of Pharmacy, Hangzhou Normal University Hangzhou School of Medicine, Hangzhou, Zhejiang, China; 4 Department of Hepatology, Hangzhou Normal University Affiliated Hospital, Hangzhou, Zhejiang, China

**Keywords:** dendritic cells, immunotherapy, immunogenicity, vaccine, antigens, neoplasm

## Abstract

**Background:**

Cancer vaccines are a promising strategy for cancer immunotherapy. Cancer vaccines elicits a specific cytotoxic immune response to tumor antigens. However, the efficacy of traditional peptide-based cancer vaccines is limited due to the inefficient delivery of antigens and adjuvants to dendritic cells (DCs). Therefore, it is necessary to develop a novel rationally designed cancer vaccine to maximize its desired effects.

**Methods:**

A Self-assembling Vehicle-free Multi-component Antitumor nanoVaccine (SVMAV) was constructed by using an unsaturated fatty acid docosahexaenoic acid (DHA)-conjugated antigen and R848 (a Toll-like receptor 7/8 agonist) to encapsulate stattic (a signal transducer and activator of transcription 3 inhibitor). The characteristics of SVMAV were investigated. The ability of SVMAV to promote DC functions was examined by in vitro analysis. The antitumor effects of SVMAV and its combination with antiprogrammed cell death protein 1 antibody (aPD-1) were also investigated in vivo. The potential application of SVMAV for neoantigen-targeted, personalized cancer vaccines was examined in an orthotopic hepatocellular carcinoma model.

**Results:**

The obtained SVMAV efficiently migrated into lymph nodes and primed CD8^+^ T cells for exert neoantigen-specific killing by promoting the antigen uptake by DCs, stimulating DC maturation, and enhancing antigen cross-presentation, due to the simultaneous delivery of the antigen, R848 and stattic. SVMAV could not only yield a robust antitumor effect for primary melanoma allografts, but also exert a protective effect for lung metastases. Moreover, combination treatment of SVMAV and aPD-1 exerted synergistic antitumor activity and extended the survival duration of melanoma-bearing mice. Notably, a cell line-specific neoantigen-based SVMAV was designed according to predicted neoantigens for Hepa1-6 cells to examine the potential application of SVMAV for personalized cancer vaccine. Encouragingly, neoantigen-specific SVMAV achieved stronger antitumor activity than aPD-1 in an orthotopic hepatocellular cancer model established with Hepa1-6 cells.

**Conclusions:**

In summary, this study offers an efficient codelivery platform for neoantigens and immunoregulatory compounds to enhance immune responses during cancer immune therapy.

## Introduction

Immunotherapy has recently become a mainstream strategy for cancer therapy in addition to surgical, chemotherapy and radiotherapy.^1^ Various approaches to cancer immunotherapies have yielded promising results in clinical trials, such as engineered chimeric antigen receptor T cells, immune checkpoint blockade (ICB) and cancer vaccines.^2–4^ Because of the capacity to elicit the intrinsic antitumor immunity in situ against tumor-specific antigens (TSAs) without in vitro manipulation of patient-derived lymphocytes, cancer vaccines have been extensively studied for more than four decades.^5 6^

The number of high-avidity and tumor-targeting cytotoxic T lymphocytes (CTLs) is critical for the effective elimination of malignant cells during cancer immunotherapy. The activation of CTLs is highly dependent on antigen-presenting cells (APCs). Dendritic cells (DCs) are the most potent APCs, and they process and present the TSAs taken up by endocytosis on the cell surface via major histocompatibility complex class I (MHC I) to specifically activate CTLs to enhance tumor killing, a process also known as cross-presentation.^7^ The therapeutic efficacy of conventional peptide-based cancer vaccines is limited due to their short circulation time, inferior immunogenicity and inefficient antigen presentation. Therefore, it is essential to efficiently deliver TSAs to DCs to facilitate antigen cross-presentation and increase the proportion of tumor-specific CTLs in order to develop more effective cancer vaccines that elicit potent antitumor immunity.

Nanotechnology rapidly developed in the field of drug development in recent years, and nanovaccines have great potential for addressing the aforementioned challenges of conventional peptide-based vaccines. First, nanoparticles are susceptible to APC phagocytosis and lymph node (LN) retention.^8^ Second, structured nanovaccines allow th codelivery of antigens and adjuvants, which favors potent activation of antigen presentation.^9^ Third, nanotechnology may significantly improve the pharmacokinetic properties of encapsulated antigens and adjuvants, such as extending drug circulation time and retarding drug degradation.^10^

Toll-like receptors (TLRs) are important components of innate immunity. TLRs are widely expressed as membrane and cytoplasmic receptors in APCs, and TLR ligands are mainly characteristic structural molecules of bacteria or viruses, such as CpG motifs, single-stranded RNA (ssRNA) and double-stranded RNA.^11^ TLR7 and TLR8 are localized to intracellular compartments and recognize ssRNA, imidazoquinoline derivatives and guanine analogs.^11^ Imidazoquinoline derivative resiquimod (R848) is a potent TLR7/8 agonist that specifically activates TLR/nuclear factor kappa-B (NF-κB) signaling pathway in APCs, which induces the expression of inflammatory factors and improves the efficiency of antigen presentation.^12^ Several studies have demonstrated that R848 exhibited potent antitumor efficacy via remodeling of the intratumoral immune microenvironment.^13^ Signal transducer and activator of transcription 3 (STAT3) is a vital nuclear transcription factor that plays key roles in tumor growth and immune suppression.^14^ Many studies reported that STAT3 inhibited DC maturation and activation and promoted immune tolerance.^15 16^ Interleukin 10 (IL-10) secreted by tumor-associated fibroblasts and tumor cells suppresses DC maturation in the tumor microenvironment (TME) via activation of the STAT3 signaling pathway, which weakens antigen presentation and T cell activation.^17^ The maturity of DCs increased significantly after STAT3 deletion in a transgenic mouse model.^18^ Therefore, STAT3 inhibitors (eg, stattic) could exert antitumor potency via abrogation of TME-mediated inhibition of DC maturation.^19^ However, the exploitation of these compounds as vaccine adjuvants is not insufficient.

In this work, we designed a novel Self-assembly Vehicle-free Multi-components Antitumor nanoVaccine (SVMAV) composed of antigen peptides, R848, and stattic via simple chemical synthesis methods and demonstrated that SVMAV stimulated robust antitumor immune responses with diminished toxic side effects. SVMAV homed to LNs after subcutaneous injection and was captured by LN-residing DCs, which promoted DC maturation and antigen cross-presentation, activated CD8^+^ T cells, and eventually initiated the targeted killing of tumor cells. Moreover, this system was suitable for the delivery of personalized cancer vaccines. Together, our study provides a promising strategy for improving the therapeutic efficacy of vaccine-based cancer immunotherapy by nanotechnology.

## Materials and methods

### Cell lines and animals

B16/F10-ovalbumin (OVA) cell was a gift from A/Prof. Yuli Lin of Fudan University. Hepa1-6 and HEK293T cells were obtained from Shanghai Cell Bank of Chinese Academy of Sciences (Shanghai, China). These cell lines were maintained with DMEM (Biological Industries, Israel) supplemented with 10% fetal bovine serum (FBS) (Biological Industries, Israel), and 1% penicillin streptomycin (Biological Industries, Israel) at a humidified incubator (Thermo Fisher Scientific, USA) with 5% CO_2_ at 37°C. Bone marrow-derived DCs (BMDCs) were collected from the femurs of 8-week-old C57BL/6 mice according to an established protocol,^20^ and cultured in BMDC complete medium composed of RPMI 1640 medium (Biological Industries, Israel) supplemented with 10% FBS, 1% penicillin streptomycin, 20 ng/mL mGM-CSF (#415-ML-020, R & D Systems, USA) and 10 ng/mL mIL-4 (#214–14, Peprotech, USA). 6-week-old male C57BL/6 mice and nod-obese diabetes server combined immune deficiency (NOD SCID) mice were purchased from Hangzhou Ziyuan Experimental Animal Technology (Hangzhou, China) and tkept in a specific pathogen free (SPF) facility. We used the ARRIVE checklist when writing our report.^21^

### Preparation of the SVMAV

The specific procedure of SVMAV preparation is described in detail in online supplemental material.

10.1136/jitc-2021-003132.supp1 Supplementary data

### Characterization of SVMAV

The procedure of characterization and cytotoxicity assessment of SVMAV are described in detail in online supplemental material.

### LN analysis

OVA peptide conjugated with fluorescein isothiocyanate (FITC) at the side chain of lysine residue was used for the tracking of OVA peptide in vivo. C57BL/6 mice were vaccinated subcutaneously with phosphate buffer saline (PBS), the combination of FITC-OVA, R848 and stattic in their free forms, or FITC-OVA@SVMAV at the dose of 40 nmol/mouse for each compound at the tail base. The inguinal LNs were harvested at 6 hours, 24 hours, and 48 hours postvaccination, and the fluorescence signals of LNs were detected using a in vivo fluorescence imaging system (Shimadzu, Japan). For the analysis of antigen uptake by DCs, the inguinal LNs were resected and processed through gentle mechanical disruption 6 hours after vaccination. The LNs homogenates were then passed through nylon mesh filters to obtain single cell suspensions, and stained with anti-CD11c-PE for the analysis by flow cytometer (DxFLEX, Beckman Coulter, USA).

### BMDC preparation

The procedure of BMDC preparation is described in detail in online supplemental material.

### RNA sequencing analysis

The RNA sequencing analysis is described in detail in online supplemental material.

### BMDC antigen uptake and presentation experiments

FITC-labeled OVA and FITC-OVA@SVMAV were used for antigen uptake experiment. BMDCs were plated in 24-well plates at 2× 10^5^ per well with BMDC complete medium, and then treated with different combinations of drugs for 4 hours at the dose of 40 nmol/mL for each compound and maintained without any treatments for 20 hours. BMDCs were then collected and stained with anti-CD11c-PE (#117307, BioLegend, USA). The percentage of FITC-positive CD11c^+^ DCs was analyzed by a flow cytometer (DxFLEX, Beckman Coulter, USA).

Unlabeled OVA and OVA@SVMAV were used for antigen presentation experiment. BMDCs (2×10^5^ cells/well) were incubated with different combinations of drugs for 4 hours at the dose of 40 nmol/mL for each compound and maintained without any treatments for 20 hours. Cells were collected and stained with anti-CD11c-FITC (#117305, BioLegend, USA), anti-CD80-PE (#104707, BioLegend, USA), and anti-SIINFEKL/H-2Kb-APC (#141606, BioLegend, USA) for 30 min at room temperature and subject for flow cytometry analysis (DxFLEX, Beckman Coulter, USA).

### Cytolytic analysis of splenocytes primed by BMDCs

To assess the cytolytic capacity of CD8^+^ T induced by stimulated DCs, B16/F10-OVA cells were used as target cells. B16/F10-OVA cells were seeded to 24-well plates (2×10^5^ per well) and pretreated overnight with 5 µg/mL of mitomycin C to prevent cell proliferation. BMDCs were incubated with different combinations of drugs for 4 hours at the dose of 40 nmol/mL for each compound and maintained without any treatments for 20 hours. Subsequently, spleens from mice vaccinated with OVA (40 nM/mice) within a week were harvested and processed to obtain splenocytes with single cell suspensions. Then, the splenocytes and BMDCs were mixed at the cell number ratio of 10: 1 (BMDCs=2 × 10^5^) and added to B16/F10-OVA cells for 48 hours. Eventually, the supernatant was collected and centrifuged to remove cell debris for the measurement of lactate dehydrogenase (LDH) release by CyQUANT LDH cytotoxicity assay kit (# C20300, Thermo Fisher Scientific, USA). The cell death can be represented by the percentage of LDH release that was calculated as follows: (sample LDH – spontaneous LDH)/(total LDH- spontaneous LDH)×100%. After removing all the non-adherent cells, the viability of B16/F10-OVA remained adhered to the culture surfaces were assessed by CCK8 assay. Additionally, remained adherent cells were fixed with 4% paraformaldehyde and stained with 0.1% crystal violet at room temperature for 10 min and dried overnight for photographic imaging.

### Tumor therapeutic and prevention experiments

Murine tumor model establishment and antitumor activity evaluation of SVMAV by both prevention and therapeutic settings are described in detail in online supplemental material.

### Flow cytometry analysis of tumor tissues

The procedure of flow cytometry analysis for tumor tissues is described in detail in online supplemental material.

### Whole-exome sequencing and data analysis

The whole-exome sequencing and data analysis are described in detail in online supplemental material.

### Therapeutic evaluation in murine orthotopic hepatocellular carcinoma model

Murine orthotopic hepatocellular carcinoma (HCC) model establishment and antitumor activity evaluation of SVMAV by therapeutic settings is described in detail in online supplemental material.

### Multiplex fluorescent immunohistochemistry for immune cell markers in HCC

The procedure of multiplex fluorescent immunohistochemistry (IHC) for immune cell markers in HCC is described in detail in online supplemental material.

### Statistical analysis

Data were shown as means±SD Statistical significance was analyzed by Student’s t-test. Statistical differences in survival were assessed by log-rank test. *P<0.05, **p<0.01, ***p<0.001, and ****p<0.0001 were taken as statistically significant. Statistical analyses were performed using GraphPad Prism V.8.0.

## Results

### Design, synthesis and characterization of SVMAV

**Figure 1:**
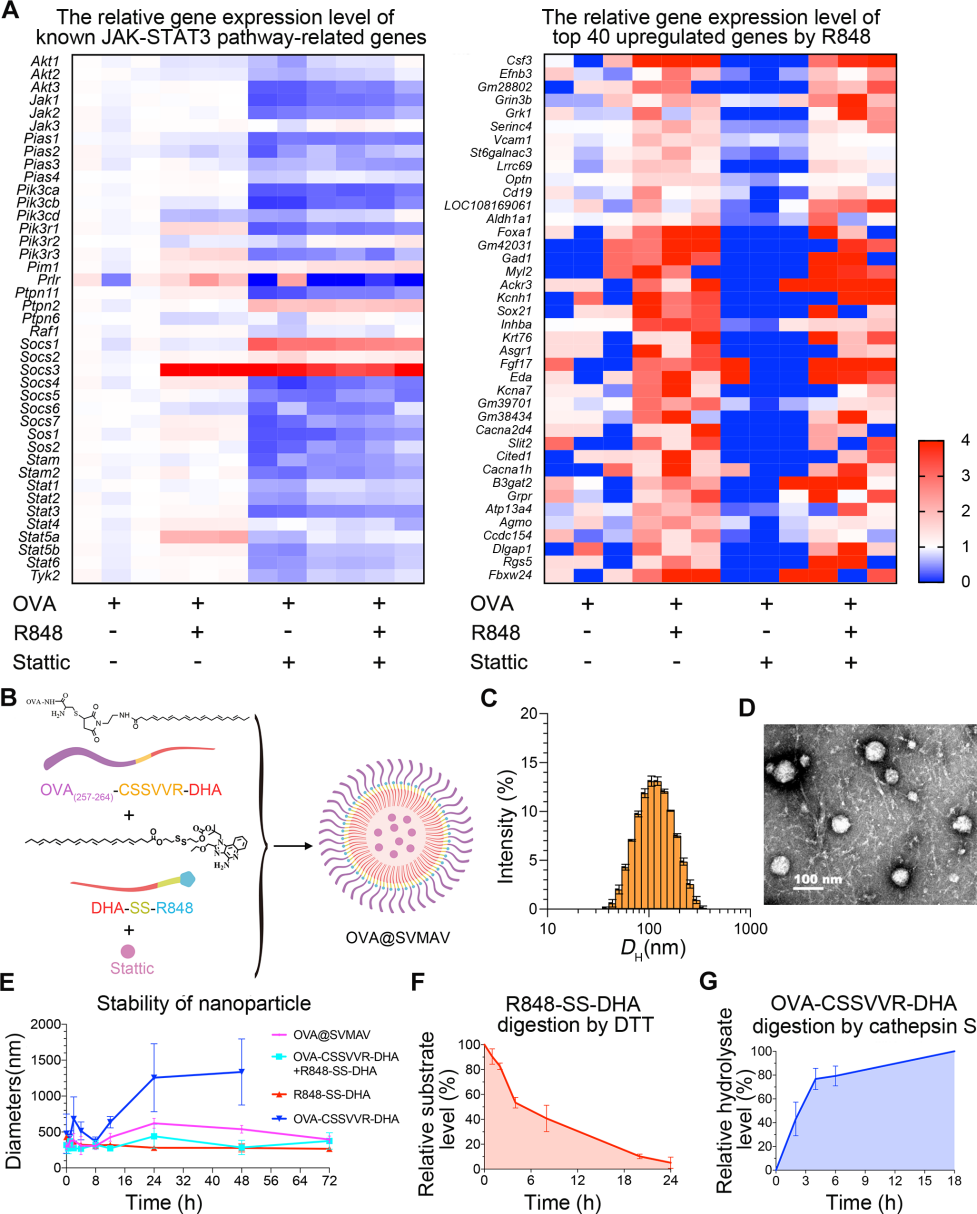
Preparation and characterization of SVMAV. (A) Heatmaps showing the gene expression levels of JAK-STAT3 pathway-related genes and the top 40 upregulated genes by R848 in BMDCs based on transcriptome sequencing results. (B) Schematic of the OVA@SVMAV preparation. (C, D) Particle diameter distribution (C) and transmission electron microscope image of OVA@SVMAV (D). (E) Stability of nanoformulations with different compositions in PBS containing 10% FBS. (F, G) Release profiles of R848 and ova from R848-SS-DHA and OVA-CSSVVR-DHA, respectively, in the simulated intracellular environment. Data are displayed as mean±SD. BMDCs, bone marrow-derived dendritic cells; DTT, dithiothreitol; FBS, fetal bovine serum; OVA, ovalbumin; PBS, phosphate buffer saline; DHA, docosahexaenoic acid; JAK, janus kinase; CSSVVR, Cys·Ser·Ser·Val·Val·Arg; STAT3, signal transducer and activator of transcription 3; SVMAV, Self-assembling Vehicle-free Multi-component Antitumor nanoVaccine.

Previous studies demonstrated that TLR activation enhanced the antigen-presentation functions of DCs, and the inhibition of STAT3 signaling promoted DC maturation. However, the combination effects of TLR activation and STAT3 inhibition have not been studied in the context of cancer immune therapies. Therefore, we treated mouse BMDCs with R848, stattic and their combination, and analyzed their transcriptome profiles by RNA sequencing to evaluate the effects of these molecules on DC functions. As expected, the combination of R848 and stattic increased the expression of immune-activating proteins, such as CSF3 and VCAM1 in BMDCs, and decreased the expression of genes involved in the suppression of DC maturation, such as STAT3 and JAK1 (figure 1A). These results suggested that these two compounds would improve the therapeutic effects of cancer vaccines via the modulation of DC functions.

With the aim of improving cancer vaccines by such additive effects of R848 and stattic, nanovaccines were constructed to simultaneously encapsulate antigen peptides, R848 and stattic. OVA _(257-264)_ (SIINFEKL) peptide was used as the antigen peptide to target B16/F10-OVA cells exogenously expressing OVA in this study. Briefly, the synthetic process of OVA@SVMAV can be divided into three steps (figure 1B, online supplemental figure 1A, B). First, the hydrophilic OVA-Cys·Ser·Ser·Val·Val·Arg (CSSVVR) peptide containing the cathepsin S response element VVR was covalently coupled to hydrophobic DHA by N- (2-aminoethyl) maleimide to yielding a controllable degradable nanopolymer monomer OVA-CSSVVR-DHA that is controllably degraded in response to cathepsin S. Then, the R848-SS-DHA containing a glutathione response element was obtained by covalently coupling hydrophilic R848 to DHA through a disulfide-bond via a reactive −OH group on R848. Finally, amphipathic OVA-CSSVVR-DHA, R848-SS-DHA and hydrophobic stattic were mixed at a molar ratio of 1: 1: 1 and spontaneously assembled into nanoparticles under the action of hydrophobicity in water via nanoprecipitation.

10.1136/jitc-2021-003132.supp2 Supplementary data

Dynamic light scattering analysis of OVA@SVMAV exhibited a narrow size distribution with a median particle diameter of ~100 nm (figure 1C). Transmission electron microscopy imaging showed that OVA@SVMAV had a uniform and spherical-like structure (figure 1D). Zeta potential analysis indicated that OVA@SVMAV had a positive zeta potential value of 40.83±1.52 mV (online supplemental figure S1E). We also evaluated the stability of the various nanoformulations in PBS containing 10% FBS at 37°C for 72 hours. The results demonstrated that the mixture of OVA-CSSVVR-DHA and R848-SS-DHA exhibited a higher stability than OVA-CSSVVR-DHA alone in a simulated body fluid environment. (figure 1E)

The drug release properties of OVA@SVMAV were analyzed by high performance liquid chromatography. R848-SS-DHA was incubated with dithiothreitol (DTT) to simulate the reductant intracellular environment, and the substrate peak areas of released R848 were measured after different durations. The results showed that R848 was steadily and effectively released via the disruption of the disulfide bond in R848-SS-DHA in the presence of DTT (figure 1F). Similarly, OVA-CSSVVR-DHA was incubated with cathepsin S, and the change in product peak areas at different time points demonstrated that cathepsin S cleaved OVA-CSSVVR-DHA to release the OVA peptide (figure 1G). Collectively, these results suggested that OVA@SVMAV was stable in a simulated body fluid environment, and its structural components could be disrupted to release functional drugs intracellularly. The cytotoxicity of OVA@SVMAV was evaluated in HEK293T cells using a CCK8 assay, and the results showed that SVMAV had a much higher IC50 than the combination of drugs in their free forms, which suggests that such nanoformulation improved their safety profile for normal epithelial cells (online supplemental figure S1C, D).

### SVMAV increased vaccine accumulation and DC uptake in LNs in vivo

**Figure 2:**
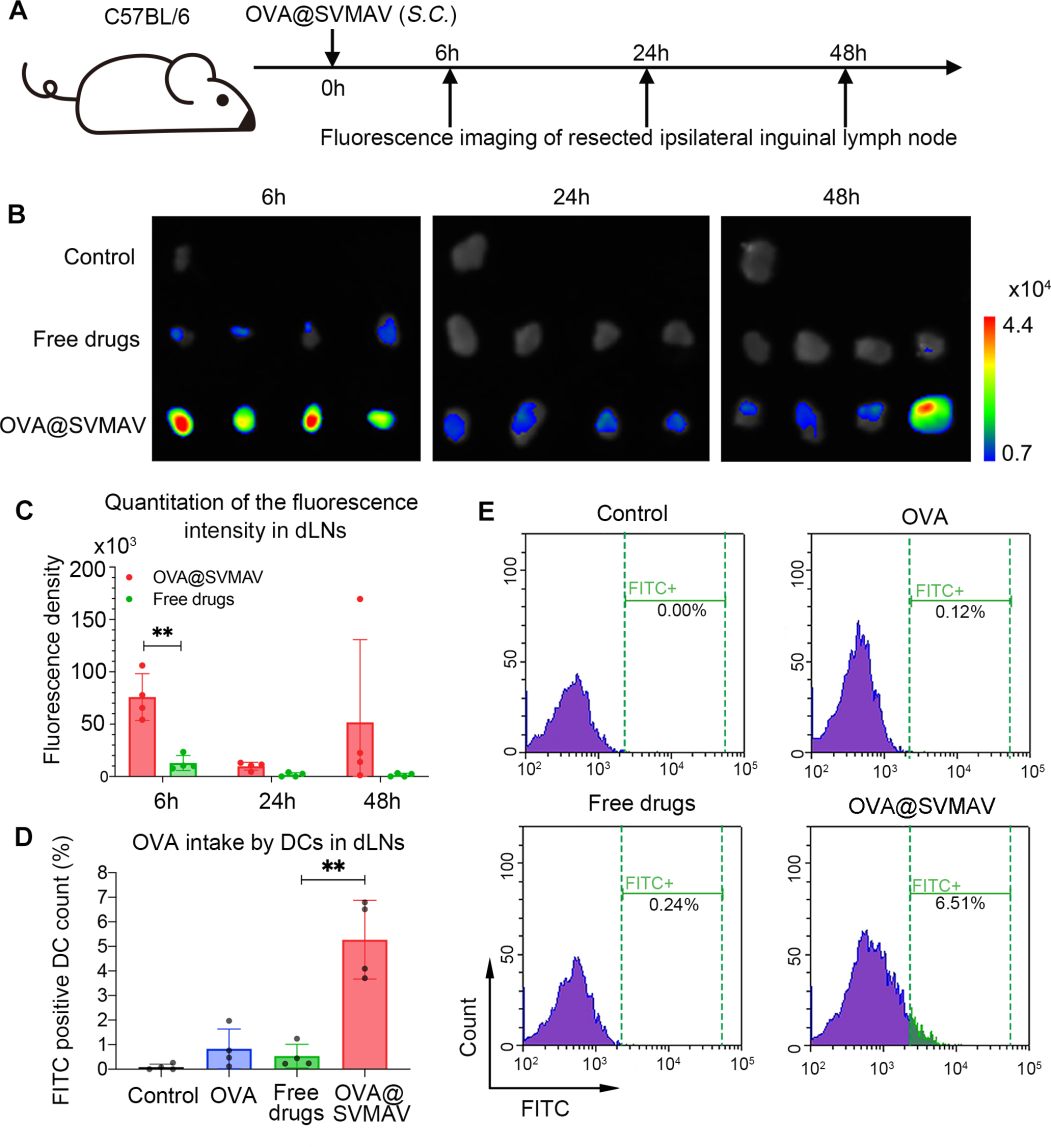
SVMAV increased vaccine accumulation and DC uptake in lymph nodes in vivo. (A) Scheme of the experimental design. (B, C) Fluorescence imaging (B) and quantification of signals (C) of draining inguinal LNs at different time points after the subcutaneous injection of different formulations of vaccines. (D, E) Quantification data (D) and representative plots (E) for the flow cytometry analysis of FITC positive LN-residing DCs at 6 hours after different treatments. Data are displayed as mean±SD. **p<0.01,. DC, dendritic cell; dLNs, draining lymph nodes; FITC, fluorescein isothiocyanate; OVA, ovalbumin; SVMAV, Self-assembling Vehicle-free Multi-component Antitumor nanoVaccine.

LNs are the major peripheral lymphatic organs within which APCs activate cytotoxic T cells after the subcutaneous administration of vaccines. Therefore, the accumulation of OVA@SVMAV in LNs was investigated in vivo using an OVA peptide labeled with FITC. After the administration of FITC-OVA +R848+ stattic in their free forms (hereafter referred to as free drugs) or FITC- OVA@SVMAV, draining inguinal LNs were harvested for fluorescence imaging at 6, 24 and 48 hours postinjection. The LNs exhibited a marginal and transient increase in FITC signal in mice treated with free drugs. In contrast, the fluorescence intensity of LNs showed a 5.8-fold increase at 6 hours postinjection in mice injected with FITC-OVA@SVMAV compared with mice treated with free drugs, and the signal was sustained until 48 hours (figure 2A–C).

In addition to vaccine accumulation in LNs, the efficiency of antigen uptake by DCs is a prerequisite for T cell activation by cross-presentation. Therefore, the uptake of FITC-OVA@SVMAV in LN-residing DCs was analyzed in mice treated with free drugs or FITC-OVA@SVMAV. Flow cytometry analysis of FITC-positive cells in CD11c^+^ DCs showed that OVA@SVMAV was taken up by 6.51% of DCs, but only 0.24% of DCs took up free drugs (figure 2D, E).

Collectively, these results showed that SVMAV promoted vaccine accumulation and antigen capture efficiency of DCs in LNs in vivo.

### SVMAV promoted DC functions in vitro

Our previous experiments showed that the R848 and stattic exhibited comprehensive influences on the intracellular signaling of DCs. Therefore, we speculated that SVMAV, which simultaneously contained these active compounds, would improve the functions of DCs. Therefore, functional analyses were performed to investigate changes in DC functions, including antigen uptake, antigen presentation and the activation of cytotoxic T cells in vitro.

**Figure 3:**
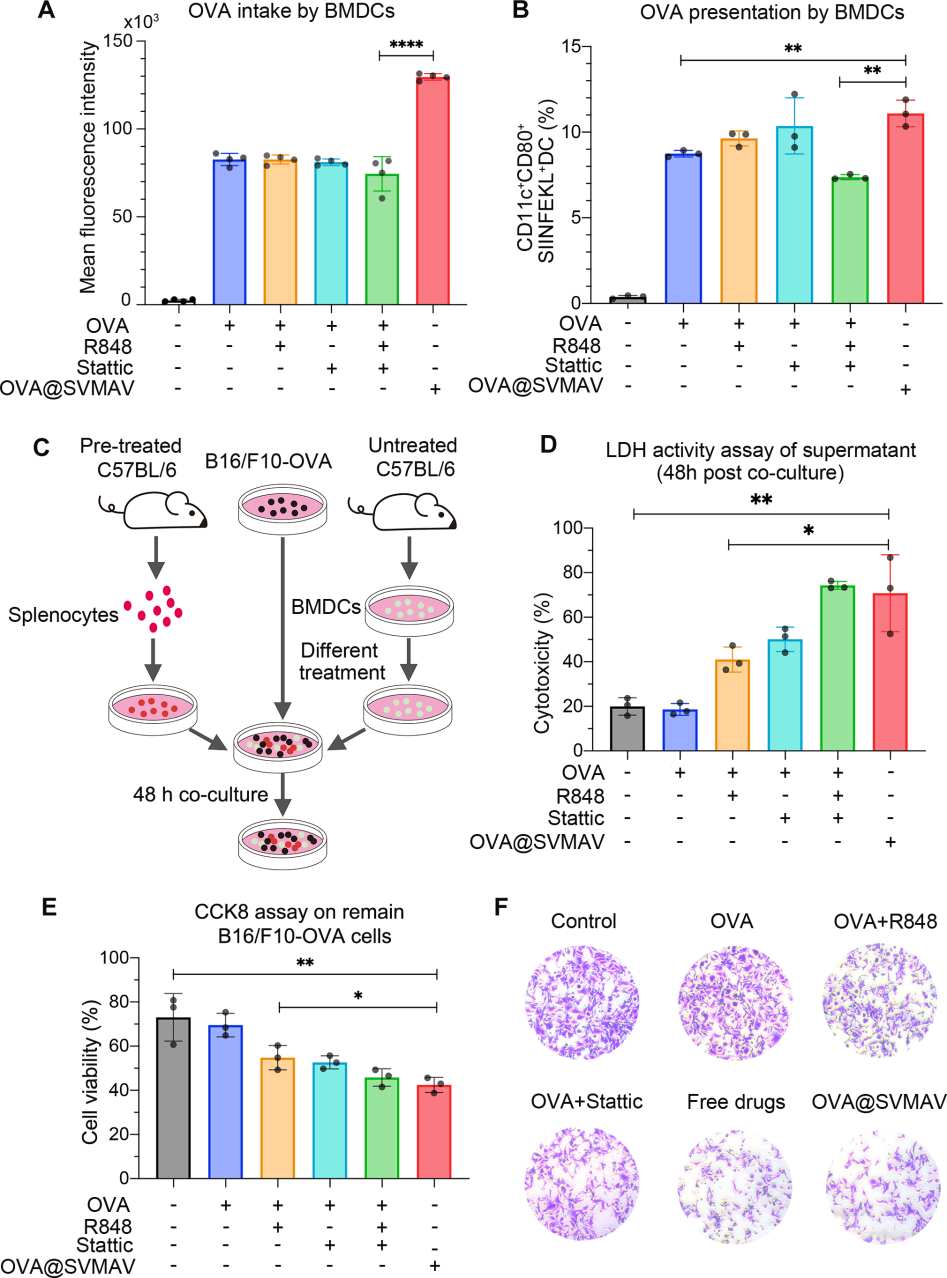
SVMAV promoted DC functions in vitro. (A) FITC fluorescence intensity of BMDCs with different treatments. (B) Frequency of CD11c^+^CD80^+^SIINFEKL^+^ BMDCs after different treatments. (C) Schematic illustration of the experimental design for cytolytic activity analysis of splenocytes primed by BMDCs. (D) The cytotoxicity of BMDCs treated with different formulations of vaccines was assessed by the LDH assays. (E) The viability of the remaining B16/F10-OVA cells was measured by the CCK8 assay. (F) Representative Photographs of the remaining B16/F10-OVA cells stained with crystal violet for different groups. Data are displayed as mean±SD. *P<0.05, **p<0.01, ****p<0.0001. BMDCs, bone marrow-derived dendritic cells; DC, dendritic cell; FITC, fluorescein isothiocyanate; LDH, lactate dehydrogenase; OVA, ovalbumin; SVMAV, self-assembling vehicle-free multicomponent antitumor nanovaccine.

BMDCs were treated with various formulations of vaccine compounds for 4 hours and maintained without any treatments for 20 hours before flow cytometry analysis to simulate the transient antigen exposure pattern in vivo. Similar to the previous results of the in vivo DC uptake analysis, in vitro OVA uptake analysis showed that BMDCs exhibited a 1.5-fold higher uptake efficiency for SVMAV compared with free drugs (figure 3A, online supplemental figure S2). Further analysis of the costimulatory molecules CD80 (a membrane marker for DC maturation) and H2kb-SIINFEKL (the antigen presentation complex) showed that the percentage of CD11c^+^CD80^+^SIINFEKL^+^ cells was significantly higher in BMDCs treated with OVA@SVMAV compared with BMDCs treated with free drugs (figure 3B). The results of these assays showed that OVA@SVMAV significantly improved antigen uptake and presentation by DCs. However, we did not observe significant improvement in these functions of BMDCs in these assays, which was likely due to the unphysiological abundance of antigens during in vitro incubation.

10.1136/jitc-2021-003132.supp3 Supplementary data

Next, we investigated whether OVA@SVMAV-treated BMDCs elicited enhanced activation of the OVA-specific CTL response in vitro by adding splenocytes from mice pulsed with the OVA peptide and BMDCs treated with various formulations of vaccine components to the B16/F10-OVA monolayer (figure 3C). Analysis of the LDH activity of these culture supernatants showed that the combination of R848 and stattic significantly increased the release of intracellular LDH to supernatants due to cell lysis by CTLs, regardless of the use of free drugs or nanovaccine (figure 3D). This observation was further confirmed in CCK8 assays and crystal violet staining assays of the remaining melanoma cells that adhered to the culture surface, which showed the lowest cell numbers in B16/F10-OVA cells coincubated with splenocytes and BMDCs treated with the combination of antigen, R848 and stattic in the forms of free drugs and nanoparticles (figure 3E, F). These results suggested that SVMAV containing R848 and stattic improved DC functions and more actively primed antigen-specific T cells for tumor-specific killing.

### SVMAV exerted potent antitumor activity by inducing a tumor antigen-specific immune response in vivo

**Figure 4:**
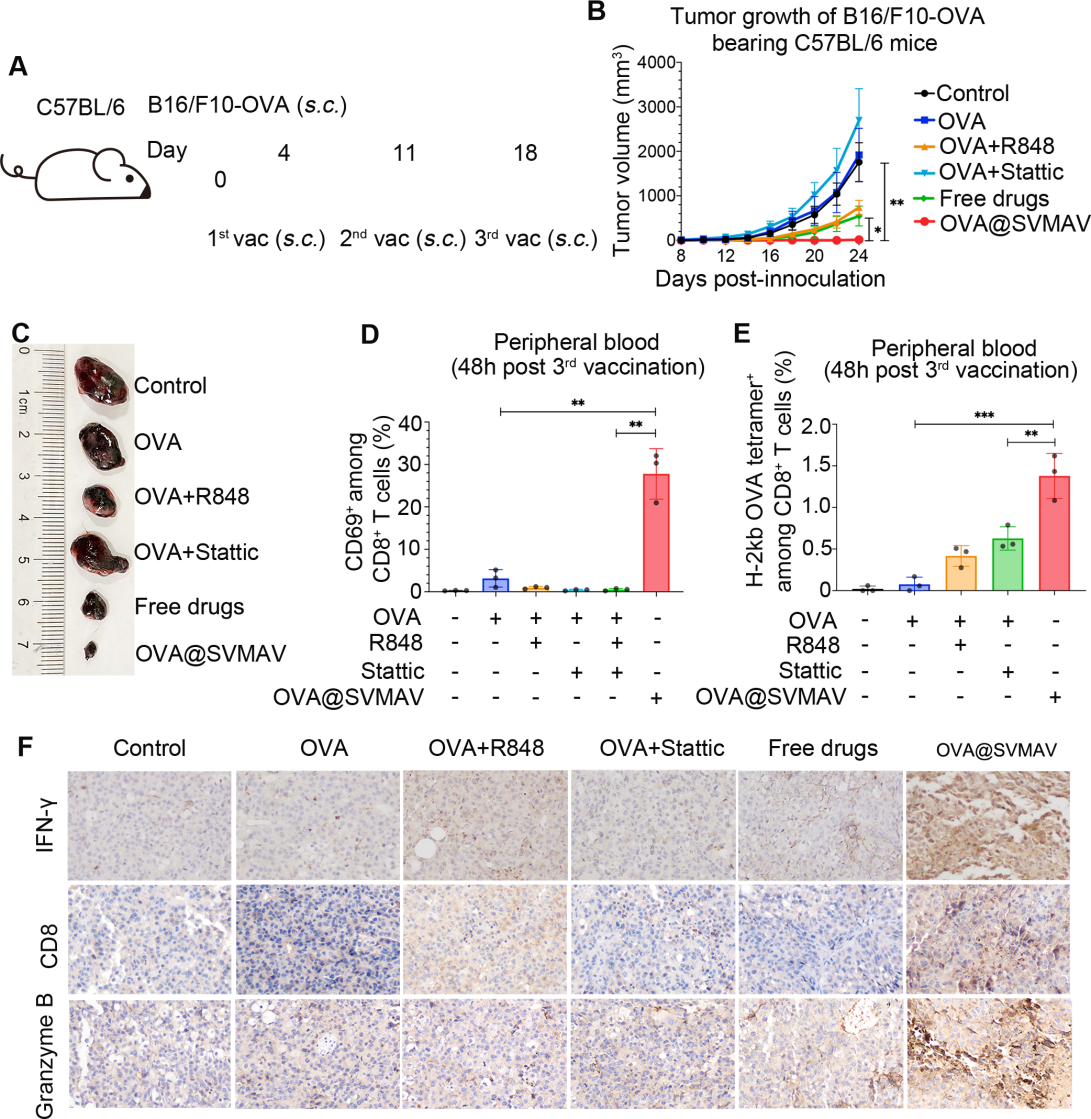
SVMAV exerted potent antitumor activity by inducing a tumor antigen-specific immune response in vivo. (A) Scheme of tumor challenge and vaccine administration. (B) Tumor growth curves for each group (n=5). (C) Representative Photographs of tumors in each group on day 24. (D, E) Percentages of CD69^+^ T cells (D) and H-2Kb ova tetramer^+^ T cells (E) in the CD8^+^ T cell population in peripheral blood from tumor-bearing mice treated with various vaccine formulations (n=3). (F) Representative Photographs of IHC for IFN-γ, CD8a, and granzyme B in tumor tissues from different groups (magnification, ×200). Data are displayed as mean±SD black scale bar represents 50 µm. *P<0.05, **p<0.01, ***p<0.001. IFN-γ, interferon γ; IHC, immunohistochemistry; OVA, ovalbumin; SVMAV, Self-assembling Vehicle-free Multi-component Antitumor nanoVaccine.

To further evaluate the antitumor efficacy of OVA@SVMAV in vivo, a subcutaneous melanoma model of B16/F10-OVA was established in immunocompetent C57/B6 mice (figure 4A). The mice were treated with various formulations of vaccine components three times at intervals of 1 week. Treatment with the OVA peptide in its free form did not obviously reduce tumor growth, but the combination of OVA, R848 and stattic significantly suppressed tumor growth, which was further enhanced by OVA@SVMAV (figure 4B, C). The stability of mouse body weight also indicated a low systemic toxicity of OVA@SVMAV (online supplemental figure S3D). Interestingly, when we repeated the same OVA@SVMAV treatment scheme in NOD SCID mice with B16/F10-OVA melanoma, OVA@SVMAV failed to exert obvious antitumor activity (online supplemental figute S3A–C), which suggests the critical role of specific immune responses during this antitumor process.

10.1136/jitc-2021-003132.supp4 Supplementary data

CTLs (CD8^+^ T cells) are essential for efficient vaccine-induced antitumor immunity. Therefore, we analyzed the status of CTLs in both peripheral blood and TME. Consistent with the differences in the antitumor properties of different formulations, analyses of the peripheral blood cells demonstrated that mice treated with OVA@SVMAV had the highest percentage of CD69^+^ cells and OVA-specific cells among peripheral CD8^+^ T cells, which indicates robust CTL activation and clonal expansion targeting the tumor antigen with the induction of SVMAV in vivo (figure 4D, E and online supplemental figure S4A, B). We also analyzed the status of CD4^+^ T cells in peripheral blood and observed strongest activation in OVA@SVMAV group, similar to our observation on peripheral CD8^+^ T cells (online supplemental figure S4C, D). IHC analysis of melanoma tissues on CTL-related markers demonstrated that mice treated with OVA@SVMAV had the highest CTL infiltration level and protein levels of interferon-γ and granzyme B, which are two important proteins secreted by CTLs to eliminate tumor cells in the TME (figure 4F and online supplemental figure S3E–G).

10.1136/jitc-2021-003132.supp5 Supplementary data

Overall, these results demonstrated that OVA@SVMAV exerted potent antitumor effects by inducing an OVA-specific CTL response.

### SVMAV reduced the pulmonary metastasis of melanoma

**Figure 5:**
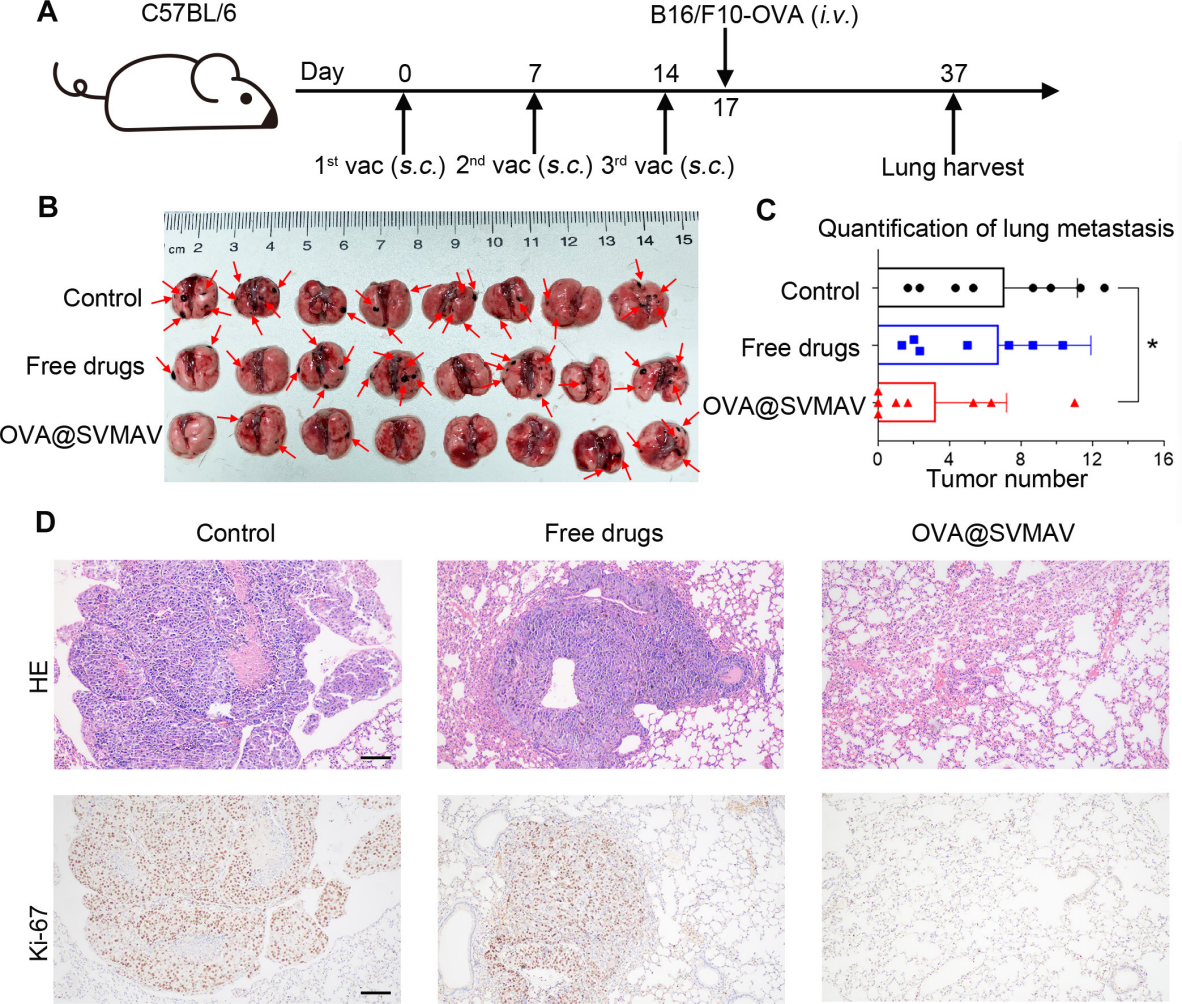
SVMAV reduced the pulmonary metastasis of melanoma. (A) Schematic of the vaccination scheme and tumor challenge. (B) Photographs of lung tissues excised on day 37; red arrows highlight metastatic foci. (C) Histogram showing the numbers of lung metastases in different groups. (D) Representative H&E staining and Ki-67 IHC staining results of lung sections from different groups. *P<0.05. Black scale bar represents 100 µm (magnification, ×100). IHC, immunohistochemistry; SVMAV, Self-assembling Vehicle-free Multi-component Antitumor nanoVaccine.

Cancer metastasis is the major cause of cancer-related mortality. Therefore, the effect of SVMAV on the prevention of cancer metastasis was evaluated with a melanoma lung metastasis model in vivo (figure 5A). C57BL/6 mice were subcutaneously treated with different formulations, including PBS, the combination of OVA, R848 and stattic as free drugs, and OVA@SVMAV once weekly for a total of three times before intravenous injection of B16/F10-OVA cells via the tail vein. Black metastatic lesions were observed on the surface of the lungs after 20 days (figure 5A). To our delight, the number of visible lung metastatic lesions was significantly decreased in mice vaccinated with OVA@SVMAV compared with the mice vaccinated with PBS or the combination of free drugs (figure 5B, C). HE and IHC for Ki-67 also showed significantly fewer micrometastatic lesions within lung tissues resected from mice vaccinated with OVA@SVMAV (figure 5D).

### SVMAV exerted synergistic antitumor effects with anti-programmed cell death protein 1 therapy

**Figure 6:**
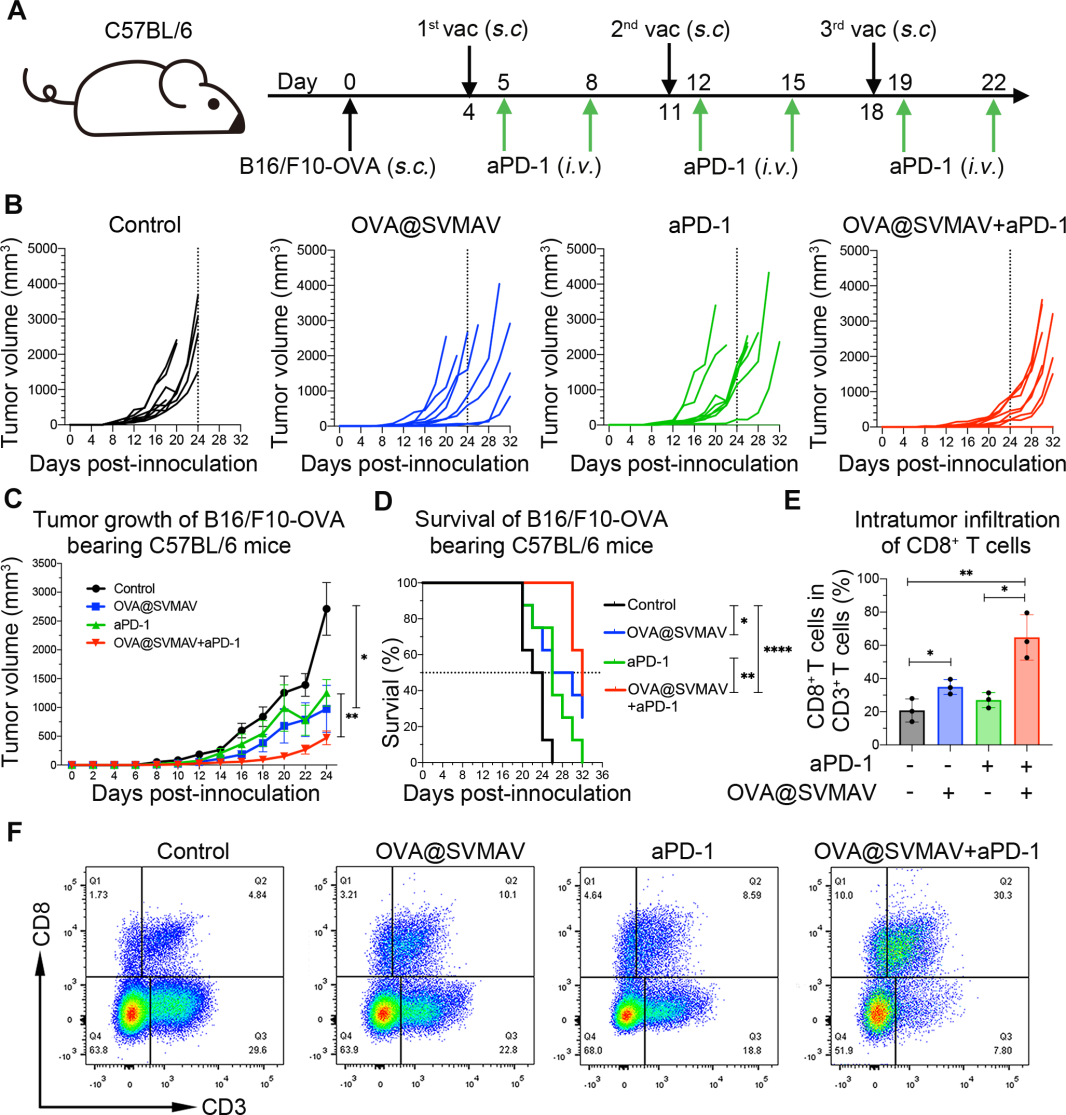
SVMAV exerted synergistic antitumor effects with aPD-1 therapy in a mouse melanoma model. (A) Scheme of tumor challenge and vaccine/aPD-1 administration. (B, C) Growth curves for individual (B) and average (C) tumor volumes in different groups (n=8). (D) Survival analysis of B16/F10-OVA tumor-bearing mice in different groups (n=8). (E) Proportions of CD8^+^ T cells among CD3^+^ T cells in tumors harvested on day 16. (F) Representative flow cytometry dot plots of CD3^+^CD8^+^ T cells in tumors harvested on day 16. Data are displayed as mean±SD. *P<0.05, **p<0.01, ****p<0.0001. aPD-1, antiprogrammed cell death protein 1; OVA, ovalbumin; SVMAV, Self-assembling Vehicle-free Multi-component Antitumor nanoVaccine.

Since tumor vaccines function by eliciting tumor-specific T-cell responses, we reasonably speculated that the therapeutic efficacy of SVMAV would be further improved in combination with ICB therapies. Therefore, the therapeutic effects of OVA@SVMAV combined with anti-programmed cell death protein 1 antibody (aPD-1) were investigated in mice bearing melanoma exogenously expressing OVA following the treatment scheme shown in figure 6A. The tumor growth curves for each tumor and the mean tumor volumes of each group showed that the monotherapies of OVA@SVMAV and aPD-1 exhibited a moderate antitumor effect, and the combination of OVA@SVMAV and aPD-1 achieved the most remarkable antitumor effect (figure 6B, C). Although the tumor volume rapidly increased after the withdrawal of treatments, the median survival durations were longer for mice treated with combination therapy (32 days) compared with untreated mice (23 days), OVA@SVMAV-treated mice (28 days) and aPD-1-treated mice (27 days) (figure 6D). One out of eight mice showed complete regression after treatment with the combination therapy of OVA@SVMAV and aPD-1. The stability of mouse body weight also indicated a low systemic toxicity of this combination therapy (online supplemental figure S6G). These results demonstrated that the combination of SVMAV and ICB therapy exerted a synergistic effect in a melanoma model.

10.1136/jitc-2021-003132.supp6 Supplementary data

To analyze whether the synergistic effect of combination therapy was attributed to the improvement of CTL infiltration in tumor, we measured the levels of intratumoral CTL infiltration during the treatment. Notably, the combination treatment of OVA@SVMAV and aPD-1 induced a significant increase in tumor-infiltrating CTLs within the TME and effectively augmented antitumor efficacy, but monotherapies with aPD-1 alone failed to elicit such a sufficient CTL infiltration (figure 6E, F). Moreover, the function of CD8^+^ T cells was also remarkedly improved by the combination therapy of OVA@SVMAV and aPD-1, as indicated by the flow cytometry analysis of IFNγ^+^ CD8^+^ T cells in tumor (online supplemental figure S5). To further evaluated the immune status in TME, the frequencies of tumor-infltrating CD4^+^ T cells, CD4^+^ Foxp3^+^ regulatory T cells (Tregs) and CD45^+^ F4/80^+^ macrophages were measured (online supplemental figure S6A, B, C, D). CD4^+^ T cells and Tregs exhibited similar levels of infiltration in all groups, but PD-1 blockade treatment decreased the tumor CD45^+^ F4/80^+^ macrophage infiltration (online supplemental figure S6C, F).

10.1136/jitc-2021-003132.supp7 Supplementary data

Overall, these results suggested that SVMAV as an effective adjunct to improve the therapeutic effect of ICB therapies, such as aPD-1, by synergistically potentiating the antitumor immunity.

### The workflow of neoantigen-targeted personalized SVMAV and the evaluation of its antitumor effect in a hepatocellular carcinoma model

**Figure 7:**
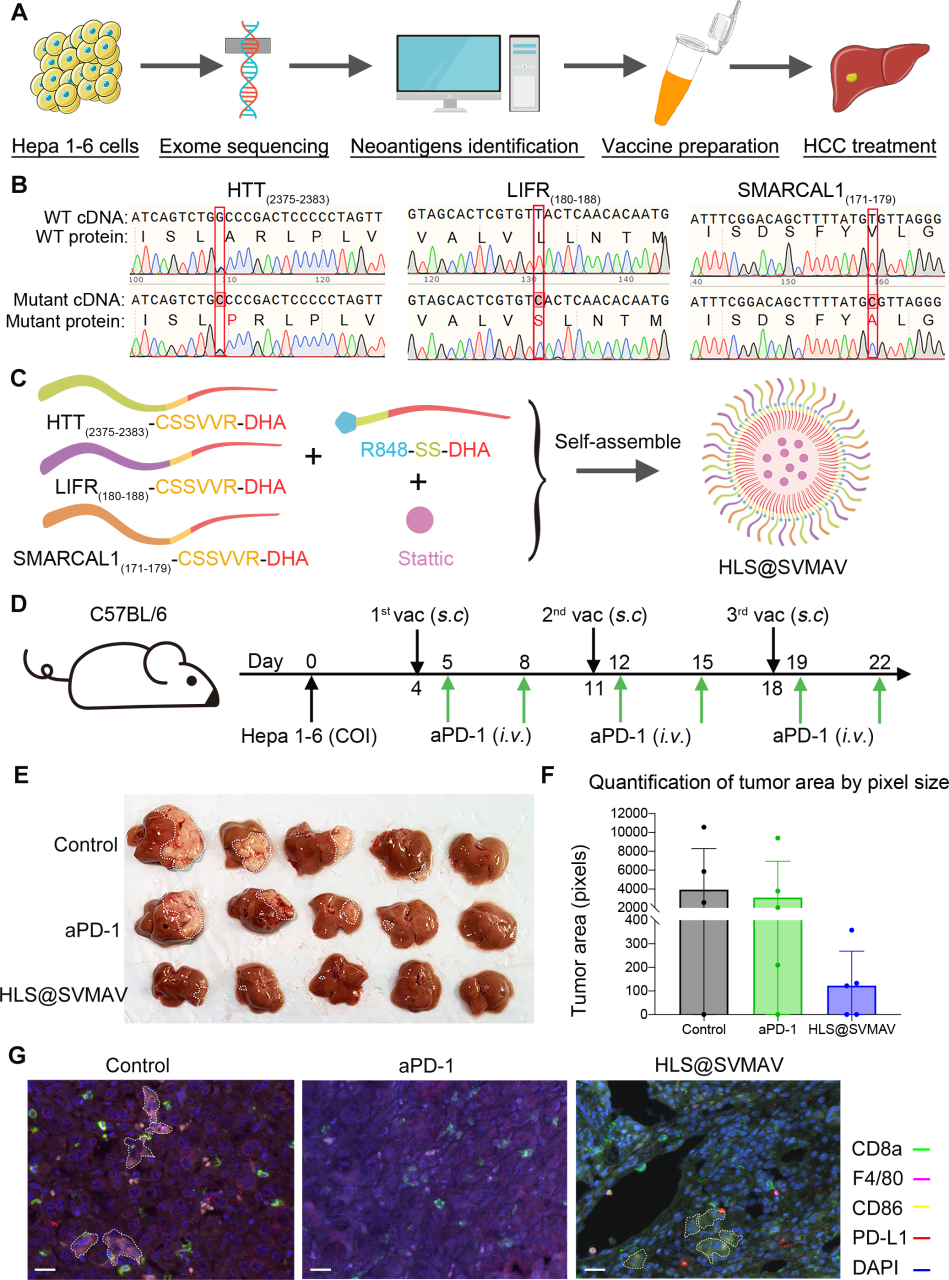
Neoantigen-targeted personalized SVMAV preparation and its antitumor effect verification. (A) Workflow of the neoantigen-targeted personalized SVMAV. (B) Prediction of three neoantigens by exome sequencing of Hepa 1–6 cells. (C) Schematic of the HLS@SVMAV preparation. (D) Treatment scheme in the orthotopic HCC mouse model. (E) Photograph of mouse livers from different groups (n=5). (F) Quantification of tumor area by pixel counting. (G) Representative multiplex fluorescent IHC staining images of orthotopic HCC tissues. CD8a, green; F4/80, magenta; CD86, yellow; PD-L1, red; DAPI, blue. white scale bar represents 20 µm. Dashed circles delimit the F4/80^+^CD86^+^ M1 macrophages. aPD-1, antiprogrammed cell death protein 1; COI, cellular orthotopic injection; HCC, hepatocellular carcinoma; IHC, immunohistochemistry; SVMAV, Self-assembling Vehicle-free Multi-component Antitumor nanoVaccine.

Taken a step further, we wanted to test whether SVMAV could be used as a universal platform for neoantigen-targeted personalized cancer vaccines in a clinical setting (figure 7A). First, we analyzed the somatic mutations of Hepa1-6, and identified potential neoantigens according to the following criteria: (1) a high binding affinity with MHC I, (2) good hydrophilicity, and (3) an exposed position for the mutated amino acid in the predicted antigen-MHC complex. After screening, we chose three eligible mutant peptides: Htt _(2375-2383)_ (ISLARLPLV → ISLPRLPLV), Lifr _(180-188)_ (VALVLLNTM → VALVSLNTM), and Smarcal1 _(171-179)_ (ISDSFYVLG → ISDSFYALG) (figure 7B). We chemically synthesized and modified these neoantigens following a previously described method and obtained HTT-CSSVVR-DHA, LIFR-CSSVVR-DHA, and SMARCAL1-CSSVVR-DHA. These modified neoantigens were mixed with R848-SS-DHA and stattic and assembled into the personalized SVMAV loaded with antigens form mutated Htt, Lifr, and Smarcal1, named HLS@SVMAV (figure 7C). To evaluate the efficacy of the designed HLS@SVMAV, an orthotopic HCC mouse model was established by inoculating Hepa1-6 cells into the left liver lobe. Mice carrying HCC cells were treated with HLS@SVMAV or aPD-1 following the treatment scheme in figure 7D. Photographic images of resected livers showed that this HCC model was highly resistant to aPD-1, which is consistent with the clinical observation that monotherapy with aPD-1 failed to improve the overall survival of HCC patients.^22–26^ To our delight, mice treated with HLS@SVMAV exhibited a significantly smaller tumor volume compared with untreated mice or aPD-1-treated mice (figure 7E, F), which suggests that neoantigen-targeted personalized SVMAV might be a promising method for HCC resistance to aPD-1 therapy.

Multiplex fluorescent IHC for CD8a, F4/80, CD86 and PD-L1 was used to evaluate the landscape of the HCC immune microenvironment to explore the mechanisms underlying the different response patterns for aPD-1 and HLS@SVMAV treatments (figure 7G). Multiplex fluorescent IHC analysis of harvested tumor tissues should be considered as images of the final event resulting from therapy-related immune response, other than immune responses during therapy. The results showed that Hepa1-6 orthotopic HCC tissues contained a high abundance of CTLs and M1 macrophages. However, aPD-1 treatment reduced the number of intratumoral F4/80^+^CD86^+^ M1 macrophages, but HLS@SVMAV did not exert a significant influence on M1 macrophages (online supplemental figure S7A, B), which is consistent with the observations in the melanoma model (online supplemental figure S6C, F). Impairment of M1 macrophages might explain the failure of aPD-1 therapy in the HCC model, and such shortcomings were overcome by HLS@SVMAV.

10.1136/jitc-2021-003132.supp8 Supplementary data

Taken together, the successful treatment of an orthotopic HCC model by neoantigen-targeted personalized SVMAV provides a proof of principle for the application of SVMAV as a universal platform for neoantigen-targeted personalized nanovaccines as an alternative strategy of cancer immune therapy.

## Discussion

The present study showed that our designed SVMAV provided a tool for the efficient codelivery of both neoantigens and synergistic adjuvants to elicit potent neoantigen-specific immune responses. Neoantigens are promising targets for cancer immunotherapy,^27–29^ which was demonstrated by the successful application of sipuleucel-T, a cancer vaccine targeting prostatic cancer-specific antigen, for the treatment of castration-resistant prostate cancer since 2010.^30 31^ However, tumor cells exert negative influences on general immune functions, such as immunosuppressive cytokines and metabolism products and ligands for immune checkpoint receptors, and naturally existing neoantigen-specific CTLs are rare in many kinds of cancers. For example, neoantigen-specific CTLs only account for less than 0.001% of the peripheral T cell population in patients with colorectal cancer.^32 33^ To solve these problems, vaccine-induced activation of tumor-specific immune responses is a highly desirable strategy, and the recent development of next-generation sequencing and bioinformatics techniques has made possible the fast identification of personalized tumor neoantigens for cancers without common neoantigens and cancers with a small number of mutations.^34^ Our nanovaccine based on the self-assembling property of polyunsaturated fatty acids, and other nanovaccines constructed by other methods, would benefit a wide range of cancer patients in the era of personalized cancer vaccines.

ICB therapies achieved remarkable therapeutic effects for the treatment of many types of cancers in recent years, including melanoma, non-small-cell lung cancer, pediatric solid tumor, renal cell carcinoma, Hodgkin lymphoma, and head and neck squamous cell carcinoma.^35 36^ However, some patients with cancer fail to respond to these therapies, and the mechanisms are not clear. Here in this study, we observed that subcutaneous melanoma responded well to a PD-1 blocking antibody, but the therapeutic effect of the same antibody in a liver cancer model was poor, which is in consistent with the observations of multiple phase II and phase III randomized clinical trials in which the objective response rate of aPD-1 monotherapy in HCC patients was only 15%–20%.^22–26^ The potential mechanisms underlying HCC resistance to ICB therapies include weak immunogenicity of HCC cells, a low infiltration level of T cells, a high infiltration level of immunosuppressive cells, and compensatory upregulation of other immune checkpoint ligands on tumor cells.^37–39^ In HCC animal models, we observed that aPD-1 treatment reduced the population of intratumoral M1 macrophages, which is known to play an important role in the antitumor immunity in liver cancer.^40 41^ This result may also partially explain the reason for the modest therapeutic efficacy of aPD-1 in HCC. Our designed HLS@SVMAV did not affect M1 macrophages, which circumvents the limitation of aPD-1 therapy in HCC. It is encouraging that SVMAV simultaneously targeting multiple HCC neoantigens exhibited significant tumor-suppressive effects via the codelivery of neoantigens and adjuvants in a rodent orthotopic HCC model, which supports its potential application in cancers that respond poorly to ICB therapies.

Other than primary cancer, we also evaluated the preventive effect of SVMAV in a metastatic melanoma model by mimicking residual circulation tumor cells via intravenous injection of tumor cells since recurrence and distant metastases are the principal causes of death of patients with cancer rather than the circumscribed growth of primary tumors.^42 43^ Therefore, adjuvant chemoradiotherapy/radiotherapy is routinely offered after tumor resection to prevent recurrence or distant metastasis.^44–46^ However, the improvements in overall clinical outcomes and quality of life were still unsatisfactory because of a high probability of developing chemoresistance and poor adherence to treatment schemes due to severe side effects. Here, we demonstrated that prophylactic application of SVMAV prevented lung metastasis with low systemic toxicity, which suggests that the vaccination targeting neoantigens may be an ideal approach for postsurgery adjuvant treatment. In addition, the surgically resected tumor itself also provides ample samples for genetic analysis, which greatly facilitates the identification of tumor neoantigens.

Personalized neoantigen-based cancer vaccines represent a paradigmatic example of precision medicine, but several challenges remain. One major limitation is the long preparation period due to dependence on sequencing analysis and peptide synthesis.^47 48^ A potential solution is the construction of a peptide library for common mutation-associated neoantigens based on a large database of cancer biobanks. With this method, personalized cancer vaccines may be readily assembled on completion of sequencing analysis is completed. The restricted pool of antigens also allows the evaluations of potential systemic toxicity and immunogenicity in advance. Therefore, novel methods for the identification of frequent cancer neoantigens and the effective assembly of highly immunogenic vaccines would accelerate the translation of personalized neoantigen-based cancer vaccines from basic science to the clinical setting.

In summary, we designed and manufactured a novel cancer nanovaccine named SVMAV that simultaneously loaded antigenic peptides, the TLR7/8 agonist R848 and the small molecule STAT3 inhibitor stattic. SVMAV enhanced vaccine accumulation in draining LNs, promoted cellular uptake of antigens, facilitated the maturation of DCs and enhanced antigen cross-presentation. SVMAV significantly inhibited the tumor growth and effectively prevented the distant metastasis. Moreover, antitumor effects of ICB therapies were remarkably improved by the combination with SVMAV. Importantly, we demonstrated that SVMAV may be a potential platform for personalized neoantigen-based cancer vaccines. Taken together, the present study provides a promising strategy for the treatment of cancer using nanovaccines.

10.1136/jitc-2021-003132.supp9 Supplementary data

## Data Availability

All data relevant to the study are included in the article or uploaded as online supplemental information. All data relevant to the study are included in the article or uploaded as supporting information.

## References

[1] Kelly PN. The cancer immunotherapy revolution. Science 2018;359:1344–5. 10.1126/science.359.6382.1344 29567702

[2] Li L, Goedegebuure SP, Gillanders WE. Preclinical and clinical development of neoantigen vaccines. Ann Oncol 2017;28:xii11–17. 10.1093/annonc/mdx681 29253113 PMC5834106

[3] Ribas A, Wolchok JD. Cancer immunotherapy using checkpoint blockade. Science 2018;359:1350–5. 10.1126/science.aar4060 29567705 PMC7391259

[4] Labanieh L, Majzner RG, Mackall CL. Programming CAR-T cells to kill cancer. Nat Biomed Eng 2018;2:377–91. 10.1038/s41551-018-0235-9 31011197

[5] Martinez-Piñeiro JA, Muntañola P. Nonspecific immunotherapy with BCG vaccine in bladder tumors: a preliminary report. Eur Urol 1977;3:11–22. 10.1159/000472047 837949

[6] van der Burg SH, Arens R, Ossendorp F, et al. Vaccines for established cancer: overcoming the challenges posed by immune evasion. Nat Rev Cancer 2016;16:219–33. 10.1038/nrc.2016.16 26965076

[7] Palucka K, Banchereau J. Cancer immunotherapy via dendritic cells. Nat Rev Cancer 2012;12:265–77. 10.1038/nrc3258 22437871 PMC3433802

[8] Blank F, Stumbles PA, Seydoux E, et al. Size-dependent uptake of particles by pulmonary antigen-presenting cell populations and trafficking to regional lymph nodes. Am J Respir Cell Mol Biol 2013;49:67–77. 10.1165/rcmb.2012-0387OC 23492193

[9] Cordeiro AS, Alonso MJ, de la Fuente M. Nanoengineering of vaccines using natural polysaccharides. Biotechnol Adv 2015;33:1279–93. 10.1016/j.biotechadv.2015.05.010 26049133 PMC7127432

[10] Ni Q, Zhang F, Liu Y, et al. A bi-adjuvant nanovaccine that potentiates immunogenicity of neoantigen for combination immunotherapy of colorectal cancer. Sci Adv 2020;6:eaaw6071. 10.1126/sciadv.aaw6071 32206706 PMC7080439

[11] Blasius AL, Beutler B. Intracellular toll-like receptors. Immunity 2010;32:305–15. 10.1016/j.immuni.2010.03.012 20346772

[12] Lee M, Park C-S, Lee Y-R, et al. Resiquimod, a TLR7/8 agonist, promotes differentiation of myeloid-derived suppressor cells into macrophages and dendritic cells. Arch Pharm Res 2014;37:1234–40. 10.1007/s12272-014-0379-4 24748512

[13] Michaelis KA, Norgard MA, Zhu X, et al. The TLR7/8 agonist R848 remodels tumor and host responses to promote survival in pancreatic cancer. Nat Commun 2019;10:4682. 10.1038/s41467-019-12657-w 31615993 PMC6794326

[14] Yu H, Lee H, Herrmann A, et al. Revisiting STAT3 signalling in cancer: new and unexpected biological functions. Nat Rev Cancer 2014;14:736–46. 10.1038/nrc3818 25342631

[15] Cheng F, Wang H-W, Cuenca A, et al. A critical role for STAT3 signaling in immune tolerance. Immunity 2003;19:425–36. 10.1016/S1074-7613(03)00232-2 14499117

[16] Wang T, Niu G, Kortylewski M, et al. Regulation of the innate and adaptive immune responses by STAT-3 signaling in tumor cells. Nat Med 2004;10:48–54. 10.1038/nm976 14702634

[17] Wang Y, Shen Y, Wang S, et al. The role of STAT3 in leading the crosstalk between human cancers and the immune system. Cancer Lett 2018;415:117–28. 10.1016/j.canlet.2017.12.003 29222039 PMC5748258

[18] Park S-J, Nakagawa T, Kitamura H, et al. Il-6 regulates in vivo dendritic cell differentiation through STAT3 activation. J Immunol 2004;173:3844–54. 10.4049/jimmunol.173.6.3844 15356132

[19] Kortylewski M, Kujawski M, Wang T, et al. Inhibiting STAT3 signaling in the hematopoietic system elicits multicomponent antitumor immunity. Nat Med 2005;11:1314–21. 10.1038/nm1325 16288283

[20] Lutz MB, Kukutsch N, Ogilvie AL, et al. An advanced culture method for generating large quantities of highly pure dendritic cells from mouse bone marrow. J Immunol Methods 1999;223:77–92. 10.1016/S0022-1759(98)00204-X 10037236

[21] Percie du Sert N, Hurst V, Ahluwalia A, et al. The ARRIVE guidelines 2.0: updated guidelines for reporting animal research. Br J Pharmacol 2020;177:3617–24. 10.1111/bph.15193 32662519 PMC7393194

[22] El-Khoueiry AB, Sangro B, Yau T, et al. Nivolumab in patients with advanced hepatocellular carcinoma (CheckMate 040): an open-label, non-comparative, phase 1/2 dose escalation and expansion trial. Lancet 2017;389:2492–502. 10.1016/S0140-6736(17)31046-2 28434648 PMC7539326

[23] Zhu AX, Finn RS, Edeline J, et al. Pembrolizumab in patients with advanced hepatocellular carcinoma previously treated with sorafenib (KEYNOTE-224): a non-randomised, open-label phase 2 trial. Lancet Oncol 2018;19:940–52. 10.1016/S1470-2045(18)30351-6 29875066

[24] Finn RS, Ryoo B-Y, Merle P, et al. Pembrolizumab as second-line therapy in patients with advanced hepatocellular carcinoma in KEYNOTE-240: a randomized, double-blind, phase III trial. J Clin Oncol 2020;38:193–202. 10.1200/JCO.19.01307 31790344

[25] Finn RS, Ryoo B-Y, Merle P, et al. Results of KEYNOTE-240: phase 3 study of pembrolizumab (Pembro) vs best supportive care (BSC) for second line therapy in advanced hepatocellular carcinoma (HCC). JCO 2019;37:4004. 10.1200/JCO.2019.37.15_suppl.4004

[26] Yau T, Park JW, Finn RS, et al. CheckMate 459: a randomized, multi-center phase III study of nivolumab (NIVO) vs sorafenib (SOR) as first-line (1L) treatment in patients (PTS) with advanced hepatocellular carcinoma (aHCC). Ann Oncol 2019;30:v874–5. 10.1093/annonc/mdz394.029

[27] Carreno BM, Magrini V, Becker-Hapak M, et al. Cancer immunotherapy. A dendritic cell vaccine increases the breadth and diversity of melanoma neoantigen-specific T cells. Science 2015;348:803–8. 10.1126/science.aaa3828 25837513 PMC4549796

[28] Gubin MM, Zhang X, Schuster H, et al. Checkpoint blockade cancer immunotherapy targets tumour-specific mutant antigens. Nature 2014;515:577–81. 10.1038/nature13988 25428507 PMC4279952

[29] McGranahan N, Furness AJS, Rosenthal R, et al. Clonal neoantigens elicit T cell immunoreactivity and sensitivity to immune checkpoint blockade. Science 2016;351:1463–9. 10.1126/science.aaf1490 26940869 PMC4984254

[30] Cheever MA, Higano CS. Provenge (Sipuleucel-T) in prostate cancer: the first FDA-approved therapeutic cancer vaccine. Clin Cancer Res 2011;17:3520–6. 10.1158/1078-0432.CCR-10-3126 21471425

[31] Higano CS, Small EJ, Schellhammer P, et al. Sipuleucel-T. Nat Rev Drug Discov 2010;9:513–4. 10.1038/nrd3220 20592741

[32] Cohen CJ, Gartner JJ, Horovitz-Fried M, et al. Isolation of neoantigen-specific T cells from tumor and peripheral lymphocytes. J Clin Invest 2015;125:3981–91. 10.1172/JCI82416 26389673 PMC4607110

[33] Alexandrov LB, Nik-Zainal S, Wedge DC, et al. Signatures of mutational processes in human cancer. Nature 2013;500:415–21. 10.1038/nature12477 23945592 PMC3776390

[34] Li L, Goedegebuure P, Mardis ER, et al. Cancer genome sequencing and its implications for personalized cancer vaccines. Cancers 2011;3:4191–211. 10.3390/cancers3044191 24213133 PMC3763418

[35] Topalian SL, Drake CG, Pardoll DM. Immune checkpoint blockade: a common denominator approach to cancer therapy. Cancer Cell 2015;27:450–61. 10.1016/j.ccell.2015.03.001 25858804 PMC4400238

[36] Wu X, Gu Z, Chen Y, et al. Application of PD-1 blockade in cancer immunotherapy. Comput Struct Biotechnol J 2019;17:661–74. 10.1016/j.csbj.2019.03.006 31205619 PMC6558092

[37] O'Donnell JS, Long GV, Scolyer RA, et al. Resistance to PD1/PDL1 checkpoint inhibition. Cancer Treat Rev 2017;52:71–81. 10.1016/j.ctrv.2016.11.007 27951441

[38] Shi T, Ma Y, Yu L, et al. Cancer immunotherapy: a focus on the regulation of immune checkpoints. Int J Mol Sci 2018;19:1389. 10.3390/ijms19051389 PMC5983802 29735917

[39] Sharma P, Hu-Lieskovan S, Wargo JA, et al. Primary, adaptive, and acquired resistance to cancer immunotherapy. Cell 2017;168:707–23. 10.1016/j.cell.2017.01.017 28187290 PMC5391692

[40] Zhang Y-L, Li Q, Yang X-M, et al. SPON2 promotes M1-like macrophage recruitment and inhibits hepatocellular carcinoma metastasis by distinct Integrin-Rho GTPase-Hippo pathways. Cancer Res 2018;78:2305–17. 10.1158/0008-5472.CAN-17-2867 29440144

[41] Tian Z, Hou X, Liu W, et al. Macrophages and hepatocellular carcinoma. Cell Biosci 2019;9:79. 10.1186/s13578-019-0342-7 31572568 PMC6761725

[42] Steeg PS. Targeting metastasis. Nat Rev Cancer 2016;16:201–18. 10.1038/nrc.2016.25 27009393 PMC7055530

[43] Siegel RL, Miller KD, Jemal A. Cancer statistics, 2020. CA Cancer J Clin 2020;70:7–30. 10.3322/caac.21590 31912902

[44] Kuipers EJ, Grady WM, Lieberman D, et al. Colorectal cancer. Nat Rev Dis Primers 2015;1:15065. 10.1038/nrdp.2015.65 27189416 PMC4874655

[45] EBCTCG (Early Breast Cancer Trialists' Collaborative Group), McGale P, Taylor C, et al. Effect of radiotherapy after mastectomy and axillary surgery on 10-year recurrence and 20-year breast cancer mortality: meta-analysis of individual patient data for 8135 women in 22 randomised trials. Lancet 2014;383:2127–35. 10.1016/S0140-6736(14)60488-8 24656685 PMC5015598

[46] Schwartz GK, Winter K, Minsky BD, et al. Randomized phase II trial evaluating two paclitaxel and cisplatin-containing chemoradiation regimens as adjuvant therapy in resected gastric cancer (RTOG-0114). J Clin Oncol 2009;27:1956–62. 10.1200/JCO.2008.20.3745 19273696 PMC2669761

[47] Ott PA, Hu Z, Keskin DB, et al. An immunogenic personal neoantigen vaccine for patients with melanoma. Nature 2017;547:217–21. 10.1038/nature22991 28678778 PMC5577644

[48] Sahin U, Derhovanessian E, Miller M, et al. Personalized RNA mutanome vaccines mobilize poly-specific therapeutic immunity against cancer. Nature 2017;547:222–6. 10.1038/nature23003 28678784

