# Supplementary material for: Self-assembly nanovaccine containing TLR7/8 agonist and STAT3 inhibitor enhances tumor immunotherapy by augmenting tumor-specific immune response
Source: J Immunother Cancer. 2021 Aug 26;9(8):e003132. doi: 10.1136/jitc-2021-003132 (PMC8404452; doi:10.1136/jitc-2021-003132)
Supplement: Supplementary data [file jitc-2021-003132supp001.pdf]

Supplementary Material

# Self-Assembly Nanovaccine Containing TLR7/8 Agonist and STAT3 Inhibitor Enhances Tumor Immunotherapy by Augmenting Tumor-Specific Immune Response.

Lele Zhang[1,2], Jiacheng Huang[1,2], Xiaona Chen[1,2], Caixu Pan[1,2], Yong He[1], Rong Su[1,2], Danjing Guo[1,2], Shengyong Yin[1,2], Shuai Wang[1,2], Lin Zhou[1,2], Jianxiang Chen[3,4*], Shusen Zheng[1,2*], Yiting Qiao[1,2*]

1. Division of Hepatobiliary and Pancreatic Surgery, Department of surgery, The First Affiliated Hospital, Zhejiang University School of Medicine, Hangzhou 310003, China;

2. NHC Key Laboratory of Combined Multi-organ Transplantation, Hangzhou 310003, China;

3. College of Pharmacy, School of Medicine，Hangzhou Normal University, Hangzhou, Zhejiang 311121, China;

4. Department of Hepatology, the Affiliated Hospital of Hangzhou Normal University; Hangzhou Normal University, Hangzhou, Zhejiang 311121, China;

* Correspondence: Yiting Qiao (yitingqiao@zju.edu.cn), Jianxiang Chen (chenjx@hznu.edu.cn) and Shusen Zheng (shusenzheng@zju.edu.cn)

Address:79# Qingchun Road, Hangzhou City 310003, Zhejiang Province, China

**Figure S1. Synthesis and characterization of SVMAV, relative to Figure 1.** (A) Synthesis of peptide-CSSVVR-DHA. (B) Synthesis of R848-SS-DHA. (C) Measurement of the cytotoxicity of the different vaccine formulations in HEK 293T cells. (D) Quantification of the IC50 levels of the different vaccine formulations. (E) Representative zeta potential distributions of OVA@SVMAV.

**Figure S2. Representative flow cytometry plots of FITC fluorescence intensity in BMDCs treated with different vaccine formulations, relative to Figure 3A.**

**Figure S3. In vivo evaluation of the therapeutic efficacy of SVMAV, relative to Figure 4.** (A) Scheme of tumor challenge and vaccine administration in NOD SCID mice. (B, C) Tumor photograph (B) and tumor growth curves (C) for B16/F10-OVA-bearing NOD SCID mice (n = 6). (D) Body weights of B16/F10-OVA-bearing C56BL/6 mice in different groups. (E) Representative photographs of IHC for INF-γ, CD8a, and Granzyme B in tumor tissues from different groups (magnification, 200×). (F, G) percentage of intratumoral granzyme $B^+$ cells. (F, G) Percentage of intratumoral granzyme $B^+$ cells (F) and $CD8^+$ T cells in nucleated cells.

**Figure S4. Influences of SVMAV on peripheral CD4$^+$ and CD8$^+$ T cells, relative to Figure 4.** (A, B, C) Representative flow cytometry plots of CD69$^+$ CD8$^+$ T cells (A), OVA-tetramer$^+$ CD8$^+$ T cells (B) and CD69$^+$ CD4$^+$ T cells (C) in peripheral blood from B16/F10-OVA-bearing C56BL/6 mice in different groups. (D) Percentages of CD69$^+$ T cells in the CD4$^+$ T cell population in peripheral blood from B16/F10-OVA-bearing mice treated with various vaccine formulations (n = 3). Please note that the data of CD4$^+$ T cells and CD8$^+$ T cells were obtained from independent bathes of experiments.

**Figure S5. Influences of SVMAV and aPD-1 on tumor infiltrating IFNγ$^+$ CD8$^+$ T cells, relative to Figure 6.**

(A) Representative flow cytometry plots of CD8$^+$ IFNγ$^+$ T cells in CD3$^+$ T cells within melanomas harvested on day 16 post inoculation (B). Representative flow cytometry plots of IFNγ$^+$ T cells in CD8$^+$ T cells. (C, D) Proportions of CD8$^+$ IFNγ$^+$ T cells in CD3$^+$ cells (C) and IFNγ$^+$ T cells in CD8$^+$ T cells (D) within the melanomas harvested on day 16 post inoculation. Please note that this batch of data and Figure 6E, F were

obtained from independent bathes of experiments.

**Figure S6. Influences of SVMAV and aPD-1 on tumor infiltrating $CD4^+$ T cells, Tregs and macrophages, relative to Figure 6.**

**(A, B, C)** Representative flow cytometry dot plots of $CD4^+$ T cells (A), $CD4^+$ $Foxp3^+$ Tregs (B) and $F4/80^+$ $CD45^+$ macrophages (C) in melanomas harvested on day 16 post inoculation. (D, E, F) Proportions of $CD4^+$ T cells in $CD3^+$ T cells (D), $Foxp3^+$ T cells in $CD4^+$ T cells (E) and $F4/80^+$ macrophages in $CD45^+$ cells (F) within the melanomas harvested on day 16 post inoculation. (G) Body weights of B16/F10-OVA-bearing C57BL/6 mice from different groups. Please note that the data of $CD4^+$ T cells/ Tregs and $CD8^+$ T cells/macrophages were obtained from different batches of mice.

**Figure S7. Quantitative assessment of MIHC on HCC model, relative to Figure 7.**

(A, B) Percentages of intratumoral $CD8^+$ T cells (A) and $F4/80^+$ $CD86^+$ M1 macrophages (B) in nucleated cells.

## Supplementary materials and methods

### Preparation of the SVMAV

**Synthesis of peptide-CSSVVR-DHA.** 2,2'-dithiobis-ethanol and docosahexaenoic acid were dissolved in dichloromethane and reacted at 43 °C for 24 h using 1- (3-dimethylaminopropyl) -3-ethylcarbodiimide as a condensation agent and N, N-diisopropylethylamine as a base. The solvent was then spun dry, extracted by liquid separation and passed through a silica gel column to obtain intermediate product DHA-mal. Next, DHA-mal and peptide-CSSVVR (Ontores Biotecknologies, China) which contained a sulfhydryl group were dissolved in dimethyl sulfoxide (DMSO) and stirred at room temperature, and the reaction progress was monitored by high performance liquid chromatography. After the reaction was completed, the end product peptide-CSSVVR-mal-DHA (referred to as peptide-CSSVVR-DHA) was obtained through lyophilizing.

**Synthesis of R848-SS-DHA.** 2,2'-dithiobis-Ethanol and docosahexaenoic acid were dissolved in dichloromethane and reacted at 43 °C for 24 h using 1-(3-dimethylaminopropyl)-3-ethylcarbodiimide as a condensation agent and 4-

dimethylaminopyridine as a base. The solvent was then spun dry, extracted by liquid separation and passed through a silica gel column to obtain intermediate product 1 DHA-SS-OH. Next, DHA-SS-OH and p-nitrophenyl chloroformate were dissolved in dichloromethane and reacted at room temperature for 8 h using N, N-diisopropylethylamine as a base. The solvent was then spun dry, extracted by liquid separation and passed through a silica gel column to obtain intermediate product 2. Finally, intermediate product 2 and R848 were dissolved in dichloromethane and reacted at 43 °C for 24 h using N, N-diisopropylethylamine as a base. The solvent was then spun dry, extracted by liquid separation and passed through a silica gel column to obtain end product R848-SS-DHA.

**Nanoparticle assembly.** Peptide-CSSVVR-DHA, R848-SS-DHA and stattic were dissolved in DMSO to give a stock solution of 6 mM/mL, respectively. Then, each stock solution was homogeneously mixed (1: 1: 1 ratio) and suspended in ddH2O (1: 9 ratio) and sonicated for 10 s.

**Characterization of OVA@SVMAV**

The morphology of the prepared nanoparticles was observed via TECNAI 10

transmission electron microscope (Philips, the Netherlands). The size distribution of nanoparticles was determined using dynamic light scattering (Malvern Instruments, UK). Zeta potential was detected using Zetasizer Nano ZS (Malvern Instruments, UK) The stability of nanoparticles was evaluated by monitoring particle size alterations in PBS (pH 7.4) with 10% FBS at 37°C for different durations. The drug release properties of OVA@SVMAV were analyzed by HPLC (Shimadzu, Japan) Briefly, R848-SS-DHA (500 μM) was incubated with DTT (5 mM) at 37 °C. OVA-CSSVVR-DHA (4mg/mL, 1 mL) was incubated with 2 μg cathepsin S at 37 °C. Aliquots (50 μL) were collected at different time points and subjected to HPLC. The substrate peak area of R848-SS-DHA at 0 h was defined as 100%, while the average product peak area of OVA-CSSVVR-DHA at 18 h was defined as 100%, then the substrate release rate at different time points could be calculated.

**Cytotoxicity assessment**

To assess the cytotoxicity of OVA@SVMAV, HEK293T cells were treated with OVA@SVMAV, R848, stattic or OVA + R848 + statiic at various concentrations, respectively. After 72 h, the cell viability was monitored using CCK8 assays

(MedChemExpress, USA) following the standard protocol.

**BMDC preparation**

Bone marrow was harvested from the femur of a C57BL/6 mouse and prepared as a single cell suspension in BMDC complete medium. BMDCs were then counted and plated on 100 mm standard sterile Petri dishes (NEST, China) at $4 \times 10^6$ in 10 mL BMDC complete medium and cultured at 37°C in 5% $CO_2$. On day 3, 10 mL fresh BMDC complete medium was added into each petri dish. On day 6 and 8, half-volume of the culture medium was replaced with fresh BMDC complete medium. On day 9, non-adherent cells (BMDC) were collected and used for subsequent experiments.

**RNA sequencing analysis**

For RNA transcriptome sequencing, BMDCs were plated in 24-well plates at $2\times 10^5$ per well with BMDC complete medium. Then the cells were treated with different combination of drugs for 2 hours at the dose of 40 nmol/mL for each compound, and collected for transcriptome sequencing.

Total RNA was extracted using the Qiagen RNeasy micro kit (74004, Qiagen, Germany) according to the manufacturer's protocol. A NanoDrop 2000 spectrophotometer

(Thermo Scientific, USA) was used to evaluated the purity and quantity of RNA. RNA integrity was determined using the Agilent 2100 Bioanalyzer (Agilent Technologies, USA). Then the libraries were constructed using NEBNext Ultra II Directional RNA and NEBNext Ultra II RNA reagents (New England Biolabs, USA) according to the manufacturer's instructions.

The libraries were sequenced on an Illumina HiSeq X Ten platform with 150 bp paired-end reads by OE Biotech Co., Ltd. (Shanghai, China). Raw reads were processed using Trimmomatic.[1] After deleting reads containing ploy N and the low quality reads from raw data, the clean reads were obtained. Then the clean reads were mapped to the mouse genome (GRCm38.p6) using HISAT2.[2] Cufflinks[3] was used to calculate FPKM 21410973) value of each gene. HTSeq-count[4] was used to generate the read counts of each gene.

To better visual presentation of RNA transcriptome sequencing data, we provide a heatmap of gene expression. The relative gene expression level was measured using the FPKM value of gene divided by the average FPKM value of gene in OVA group. The top 40 upregulated genes by R848 and known JAK-STAT3 signaling pathway-related

genes (obtained from Kyoto Encyclopedia of Genes and Genomes platform) regulated by stattic were shown in the heatmap.

**Therapeutic evaluation in murine subcutaneous melanoma model**

6-week-old male C57BL/6 mice were injected subcutaneously with $1 \times 10^5$ B16/F10-OVA cells. Subsequently, mice were treated with different combinations of drugs at the dose of 20 nM/mice for each compound by subcutaneous injecting at tail base on day 4, 11 and 18 post tumor inoculation. Tumor volume ([length × width× width]/2) was measured every 2 days by Jiacheng Huang who do not know the group allocation, and mice were euthanized when tumor volume exceeded 2000 $mm^3$. Approximately 100 μL of peripheral blood cells were collected from the inner canthus of mice at 48 hours after the last vaccination. One aliquot of blood cells was stained with anti-CD69-APC (#104513, BioLegend, USA) and anti-CD4-PE (#100408, BioLegend, USA). One aliquot of blood cells was stained with anti-CD69-APC (#104513, BioLegend, USA) and anti-CD8-PE/Cy7 (#100721, BioLegend, USA). The other aliquot was stained with anti-CD8-FITC (#100706, BioLegend, USA) and T-Select H-2Kb OVA Tetramer-SIINFEKL-APC (#TS-5001-2C, MBL, Japan). Samples were incubated with

antibodies for 30 minutes at room temperature before being analyzed by flow cytometry (DxFLEX, Beckman Coulter, USA). All mice were sacrificed on day 24, and tumor were collected.

To perform IHC staining of INF-γ， granzyme B and CD8a, mouse monoclonal antibodies of IFN-γ (#A00393-3, Boster, China), granzyme B (#ab134933, Abcam, UK) and CD8a (#A02236-1, Boster, China) were used in dilutions of 1:200, 1:200 and 1:200, respectively.

Additionally, the same subcutaneous melanoma model was also established in 6-week-old NOD SCID mice and treated with PBS or OVA@SVMAV following the same treatment scheme. Mice were sacrificed at day 22, and tumors were collected.

In order to evaluate the synergesitc effect of OVA@SVMAV with aPD-1. B16/F10-OVA cells ($1.5 \times 10^5$) were subcutaneously inoculated at the right flanks of each C57BL/6 mouse (set as day 0). The mice were randomly assigned to 4 groups and treated with PBS, OVA@SVMAV, aPD-1 (#BE0148, Biocell, USA), or the combination of OVA@SVMAV and aPD-1, respectively. OVA@SVMAV (20 nM/mouse) were subcutaneously injected on day 4,11, and 18. aPD-1 (100 μg/mouse)

were injected via tail vein on days 5, 8, 12, 15, 19, and 22. Tumor volume and body weight were monitored every 2 days by Jiacheng Huang who do not know the group allocation, and mice were euthanized when tumor volume exceeded 2000 $mm^3$.

**Flow cytometry analysis of tumor tissues**

The melanomas were collected and mechanically triturate to obtain single cell suspension. Mononuclear cells were isolated by OptiPrep Density Gradient Medium (Sigma-Aldrich, USA). One aliquot of cells was stained with anti-CD3-APC/Cy7 (#100222, BioLegend, USA) and anti-CD8-FITC (#100706, BioLegend, USA). One aliquot of cells was stained with anti-CD3-FITC (#100204, BioLegend, USA), anti-CD4-PE (#100408, BioLegend, USA) and anti-Foxp3 alexa fluor 647 (#126407, BioLegend, USA). One aliquot of cells was stained with anti-CD3-FITC (#100204, BioLegend, USA), anti-IFNγ-PE (#505808, BioLegend, USA) and anti-CD8--PE/Cy7 (#100721, BioLegend, USA) The last aliquot was stained with anti-CD45-APC/Fire™ 750 (#103153, BioLegend, USA) and anti-F4/80-PE (#123109, BioLegend, USA). Samples were incubated with antibodies for 30 minutes at room temperature before being analyzed by flow cytometry (DxFLEX, Beckman Coulter, USA).

**Tumor prevention experiments in murine melanoma lung metastasis model**

To further evaluate the metastasis inhibition effectiveness of OVA@SVMAV, C57BL/6 mice were vaccinated with different combinations of drugs at the dose of 20 nM/mice for each compound on day 0, 7 and 14 days, respectively before the injection of B16/F10-OVA cells ($1\times 10^5$) via the tail vein on day 17. At day 37, all mice were sacrificed, the lungs were harvested. Visible surface metastases were counted. Lung tissues were then processed for paraffin sections and subject to HE staining and IHC using Ki-67 antibody at 1:10000 dilution (#27309-1-AP, Proteintech, USA).

**Whole exome sequencing and data analysis**

Genomic DNA of Hepa1-6 cell was extracted. The library was prepared and sequenced using Agilent Mouse Exome reagent (Agilent Technologies, USA) following the manufacturer’s instructions. Briefly, 180 - 280 bp fragments were generated by hydrodynamic shearing system (Covaris, USA) and were ligated to adapter molecules on both ends for PCR reaction. After PCR reaction, libraries were hybridized with a biotin labeled probe in the liquid phase and then gene exons were captured by magnetic beads with streptomycin. Captured libraries were enriched by PCR reaction to add

index tags to prepare for subsequent sequencing. The products were purified using an AMPure XP system (Beckman Coulter, USA) and then quantified by an Agilent High Sensitivity DNA Assay (Agilent 2100 Bioanalyzer System, USA).

The sample clustering was performed by a cBot Cluster Generation System using Hiseq PE Cluster Kit (Illumina, USA) according to the manufacturer's recommendations. After clustering finished, the DNA libraries were sequenced on the Illumina HiSeq platform and then paired-end reads of 150 bp in length were generated.

To get the original mapping results, valid sequencing data was mapped to the mouse reference (GRCm38/mm10) by Burrows-Wheeler Aligner software[5] and the data was stored in BAM format. The somatic SNV was detected by muTect[6] and the somatic InDel by Strelka.[7]

NetMHCPan[8] was used to predict the MHCI-binding affinity of mutant peptides. The hydrophilicity of the mutant peptides was predicted by http://www.innovagen.com/proteomics-tools. 3 eligible mutant peptides were chosen as neoantigens.

**Therapeutic evaluation in murine orthotopic HCC model**

To establish the mouse orthotopic HCC model, a total of 20 μL mixture of DMEM medium and Matrigel (with the ratio of 1: 1, #356255, Corning, USA) containing 5 × $10^6$ Hepa 1-6 cells were inoculated into the left lobe of the liver of each mouse. Then, mice were randomly divided into 3 groups, and treated with PBS, HLS@SVMAV, or aPD-1, respectively. HLS@SVMAV (20 nM/mouse) were subcutaneously injected on day 4,11, and 18. aPD-1 (100 μg/mouse) were injected via tail vein on days 5, 8, 12, 15, 19, and 22. At day 30, all mice were euthanized and livers were harvest for further analysis. Tumor area was measured by pixel counting function of Image J software.

**Multiplex fluorescent IHC for immune cell markers in HCC.**

Tissue multicolor IHC staining was performed by Opal 7-Color Manual IHC Kit (NEL861001KT, PerkinElmer, USA) according to the manufacturer's instruction. Briefly, HCC tissues were fixed and processed for paraffin sections. After routinely dewaxing and rehydration, the slices were subjected to antigen retrieval and quenching of endogenous peroxidase using microwave treatment in AR6 buffer. The slices were blocked with PerkinElmer Antibody Diluent/Block buffer and incubated with the

primary antibody. Then, the slices were incubated in Opal polymer HRP Ms + Rb for 10 min at room temperature. The opal signal was amplificated by incubated the slices in Opal Working Solution. Immediately after that, the slides were heated in AR6 buffer in a microwave oven to strip the primary-secondary-HRP complete in order to introduction of the next primary antibody. After all antibody staining is completed, the slices were counterstained with 4,6-diamidino-2-phenylindole (DAPI) and covered with mounting medium (Prolong Diamond Antifade Mountant, ThermoFisher, USA). The primary antibodies were as follows: CD8a (1:3000, #ab217344, Abcam, UK), F4/80 (1:200, #ab111101, Abcam, UK), CD86 (1:100, #ab234401, Abcam, UK) and PD-L1 (1:200, #ab233482, Abcam, UK). The slices were imaged using Vectra Polaris Quantitative Pathology Imaging System.

**Reference**


1. Bolger AM, Lohse M, Usadel B. Trimmomatic: a flexible trimmer for Illumina sequence data. *Bioinformatics* 2014;30(15):2114-20. doi: 10.1093/bioinformatics/btu170 [published Online First: 2014/04/04]
2. Kim D, Langmead B, Salzberg SL. HISAT: a fast spliced aligner with low memory requirements. *Nat Methods* 2015;12(4):357-60. doi: 10.1038/nmeth.3317

[published Online First: 2015/03/10]

3. Trapnell C, Williams BA, Pertea G, et al. Transcript assembly and quantification by RNA-Seq reveals unannotated transcripts and isoform switching during cell differentiation. *Nat Biotechnol* 2010;28(5):511-5. doi: 10.1038/nbt.1621 [published Online First: 2010/05/04]
4. Anders S, Pyl PT, Huber W. HTSeq--a Python framework to work with high-throughput sequencing data. *Bioinformatics* 2015;31(2):166-9. doi: 10.1093/bioinformatics/btu638 [published Online First: 2014/09/28]
5. Li H, Durbin R. Fast and accurate short read alignment with Burrows-Wheeler transform. *Bioinformatics* 2009;25(14):1754-60. doi: 10.1093/bioinformatics/btp324 [published Online First: 2009/05/20]
6. Cibulskis K, Lawrence MS, Carter SL, et al. Sensitive detection of somatic point mutations in impure and heterogeneous cancer samples. *Nat Biotechnol* 2013;31(3):213-9. doi: 10.1038/nbt.2514 [published Online First: 2013/02/12]
7. Saunders CT, Wong WS, Swamy S, et al. Strelka: accurate somatic small-variant calling from sequenced tumor-normal sample pairs. *Bioinformatics* 2012;28(14):1811-7. doi: 10.1093/bioinformatics/bts271 [published Online First: 2012/05/15]
8. Nielsen M, Lundegaard C, Blicher T, et al. NetMHCpan, a method for quantitative predictions of peptide binding to any HLA-A and -B locus protein of known sequence. *PLoS One* 2007;2(8):e796. doi: 10.1371/journal.pone.0000796 [published Online First: 2007/08/30]
