# Supplementary figures and images for: Self-assembly nanovaccine containing TLR7/8 agonist and STAT3 inhibitor enhances tumor immunotherapy by augmenting tumor-specific immune response
Source: J Immunother Cancer. 2021 Aug 26;9(8):e003132. doi: 10.1136/jitc-2021-003132 (PMC8404452; doi:10.1136/jitc-2021-003132)

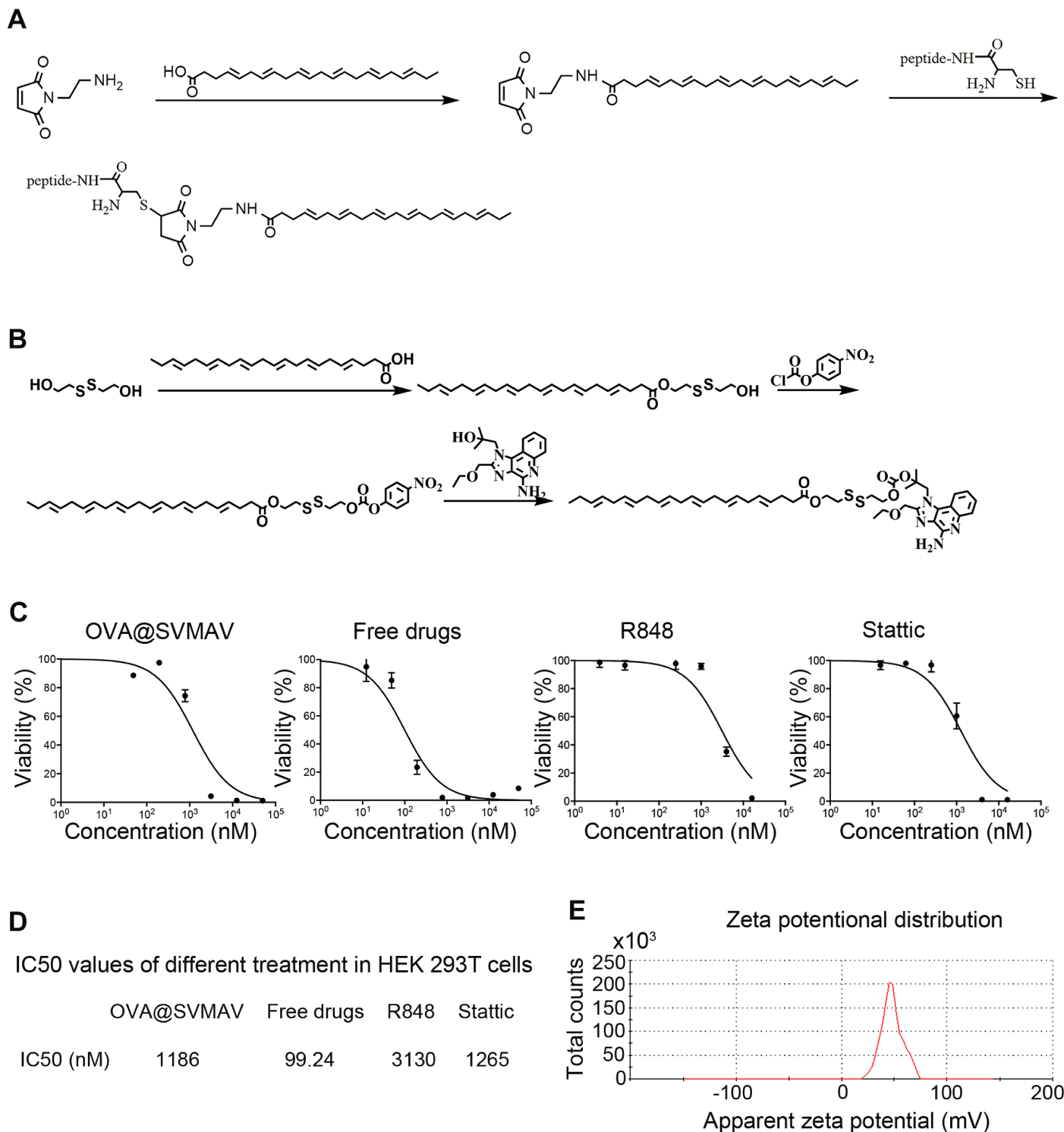

Supplement: Supplementary data [file jitc-2021-003132supp002.pdf]

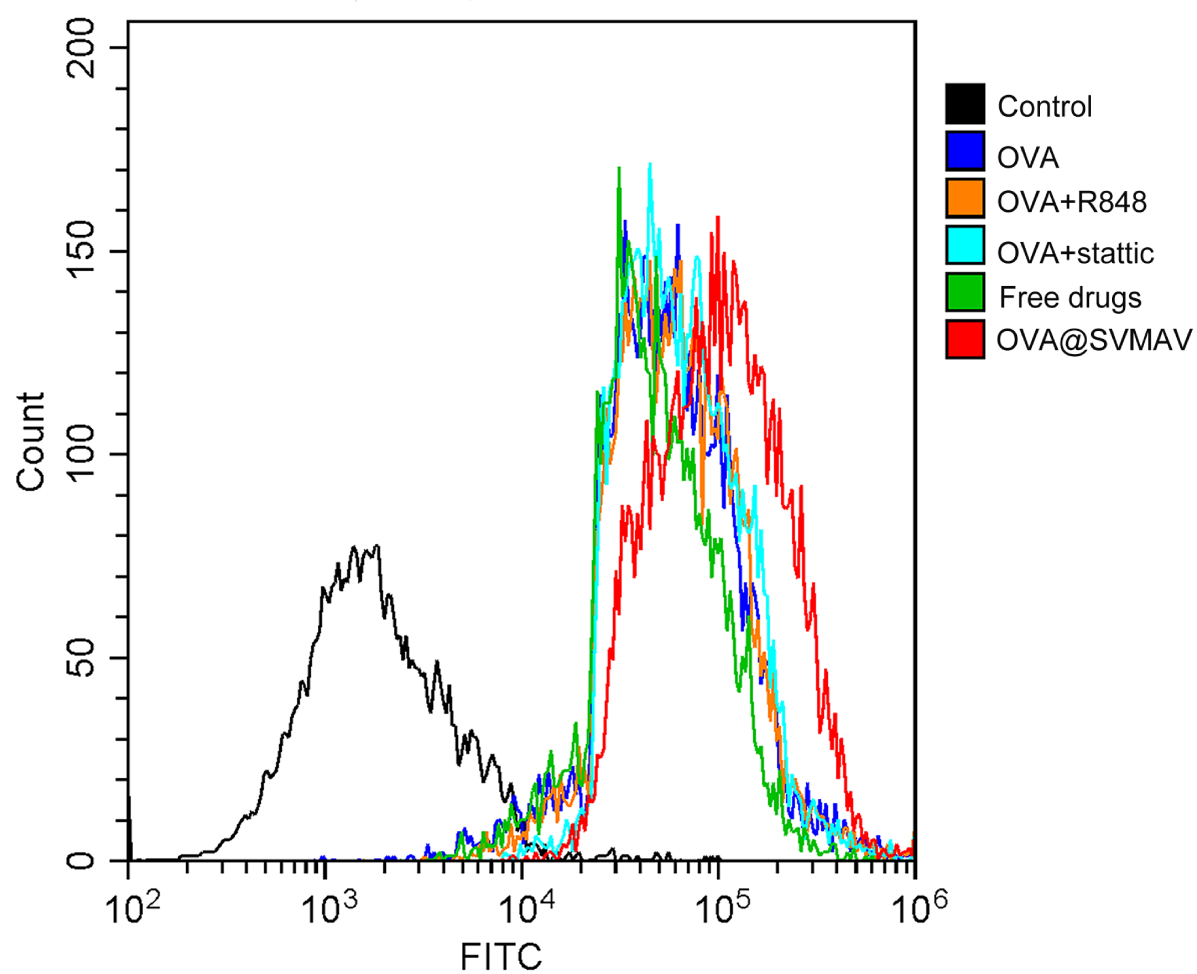

Supplement: Supplementary data [file jitc-2021-003132supp003.pdf]

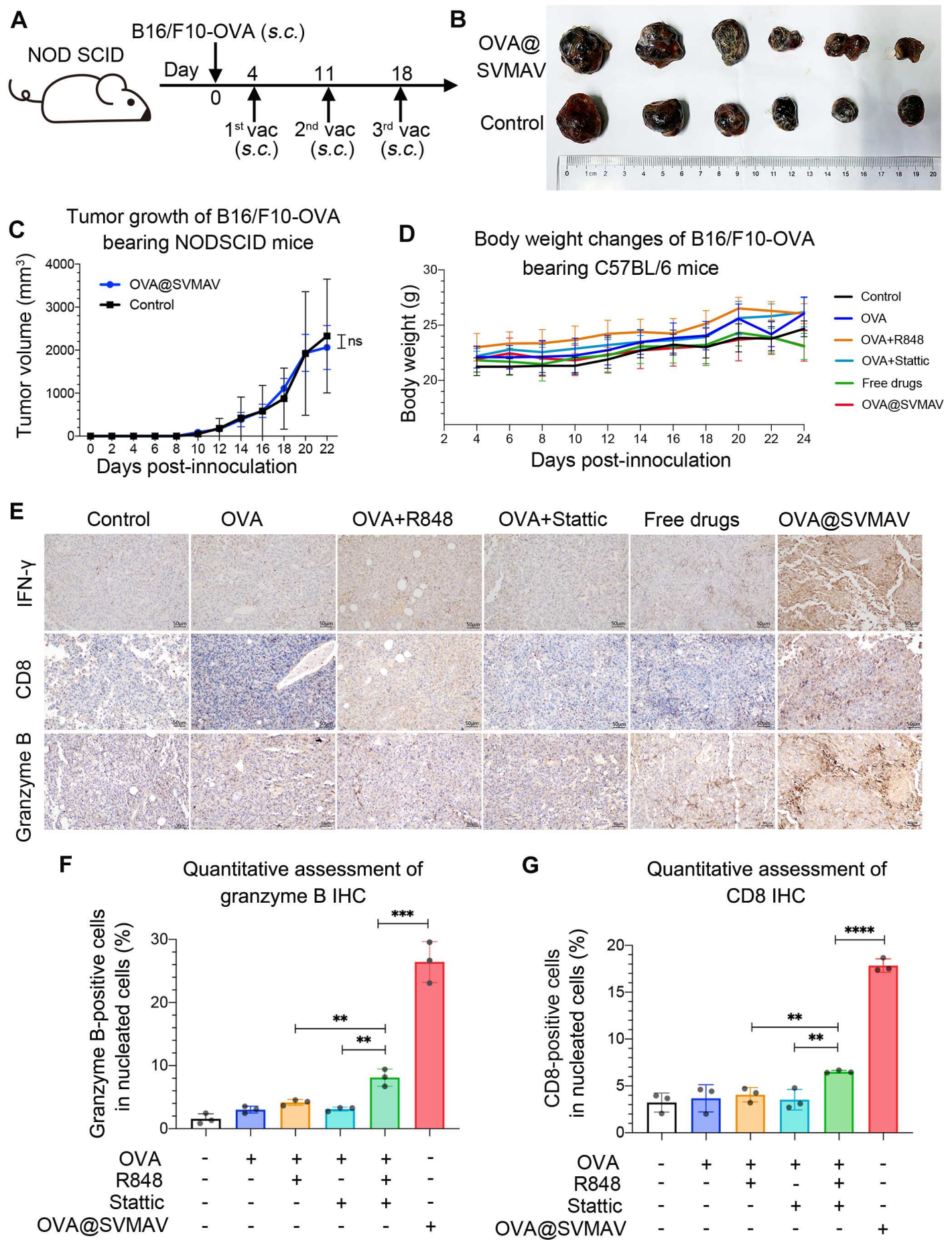

Supplement: Supplementary data [file jitc-2021-003132supp004.pdf]

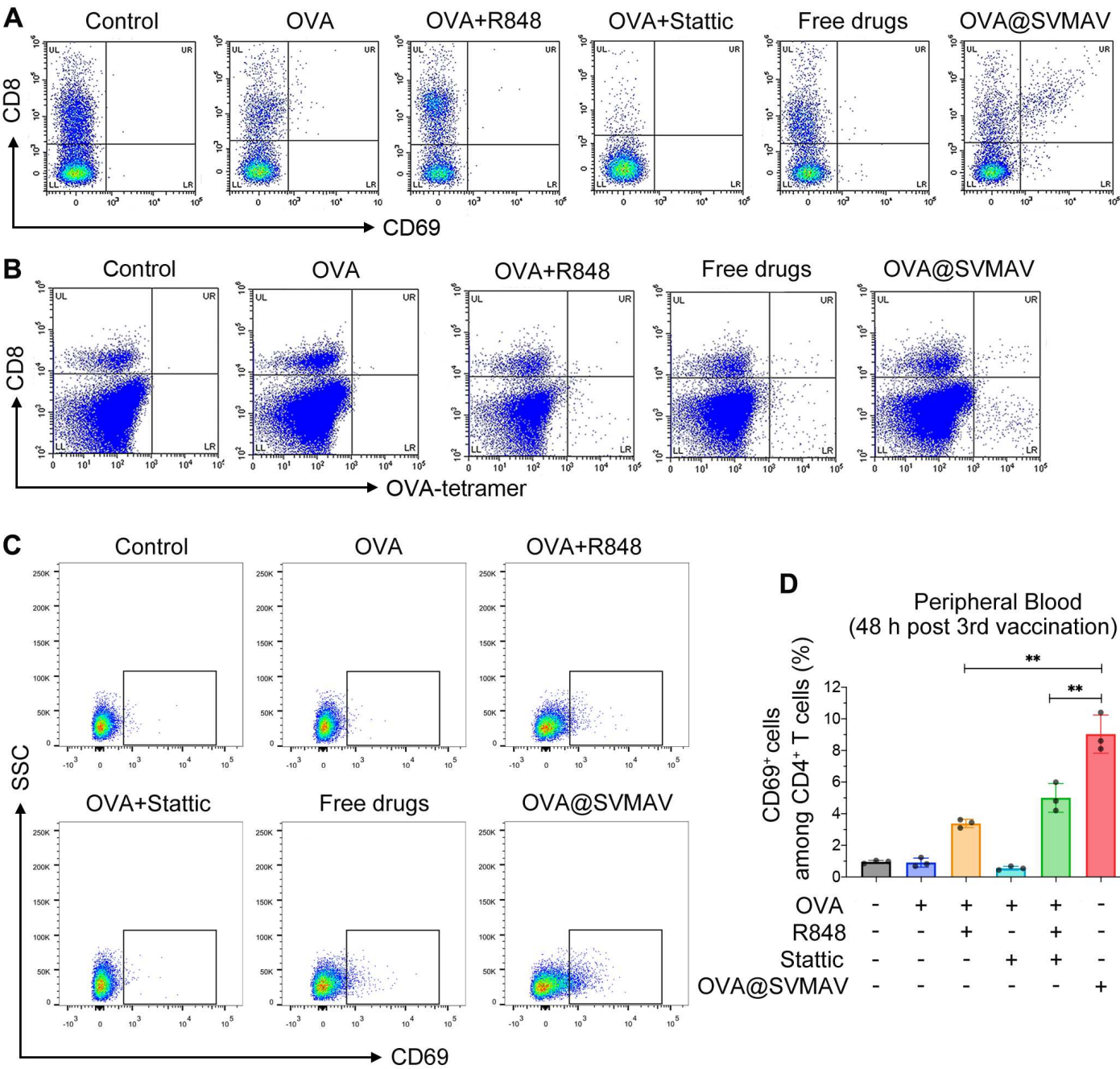

Supplement: Supplementary data [file jitc-2021-003132supp005.pdf]

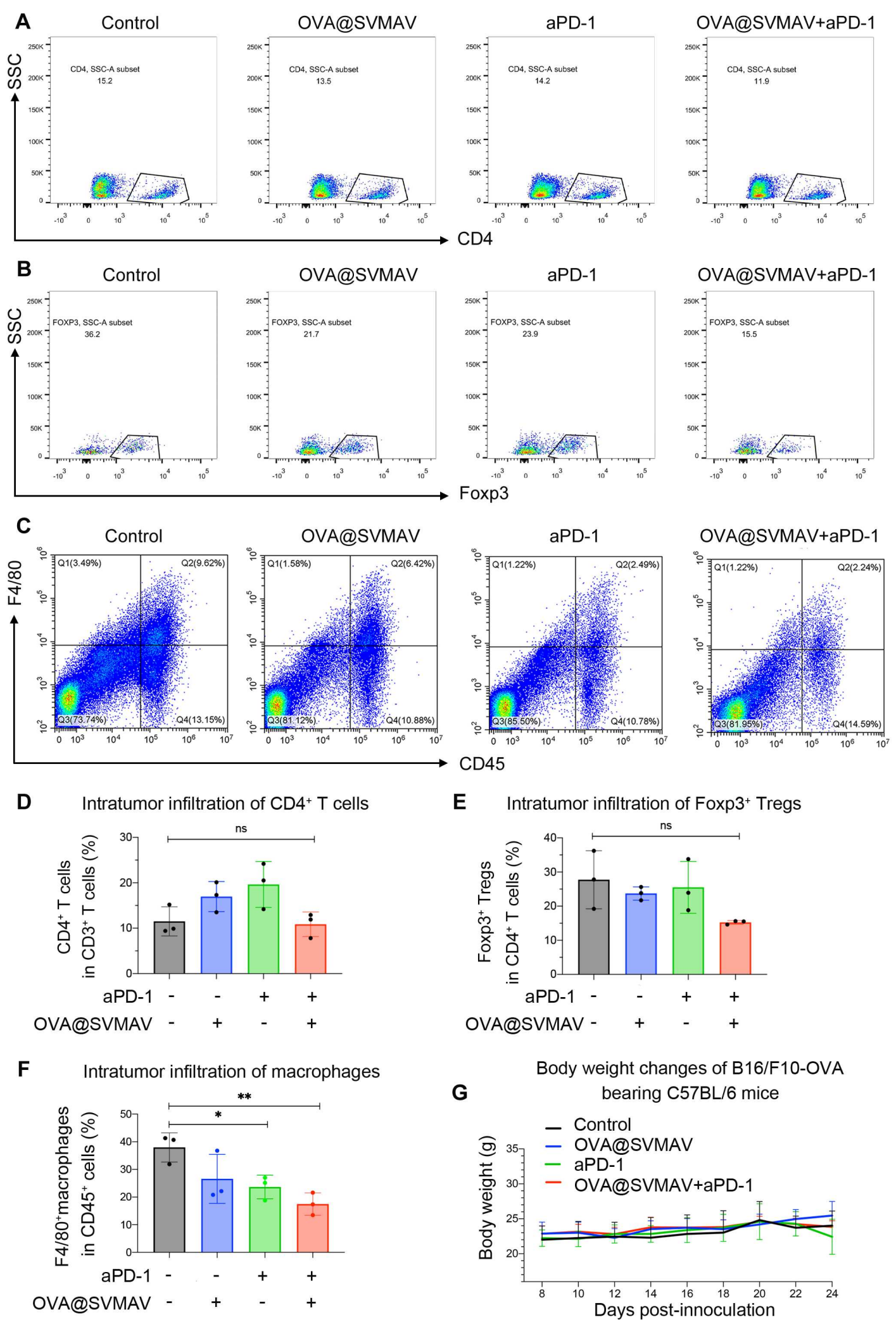

Supplement: Supplementary data [file jitc-2021-003132supp006.pdf]

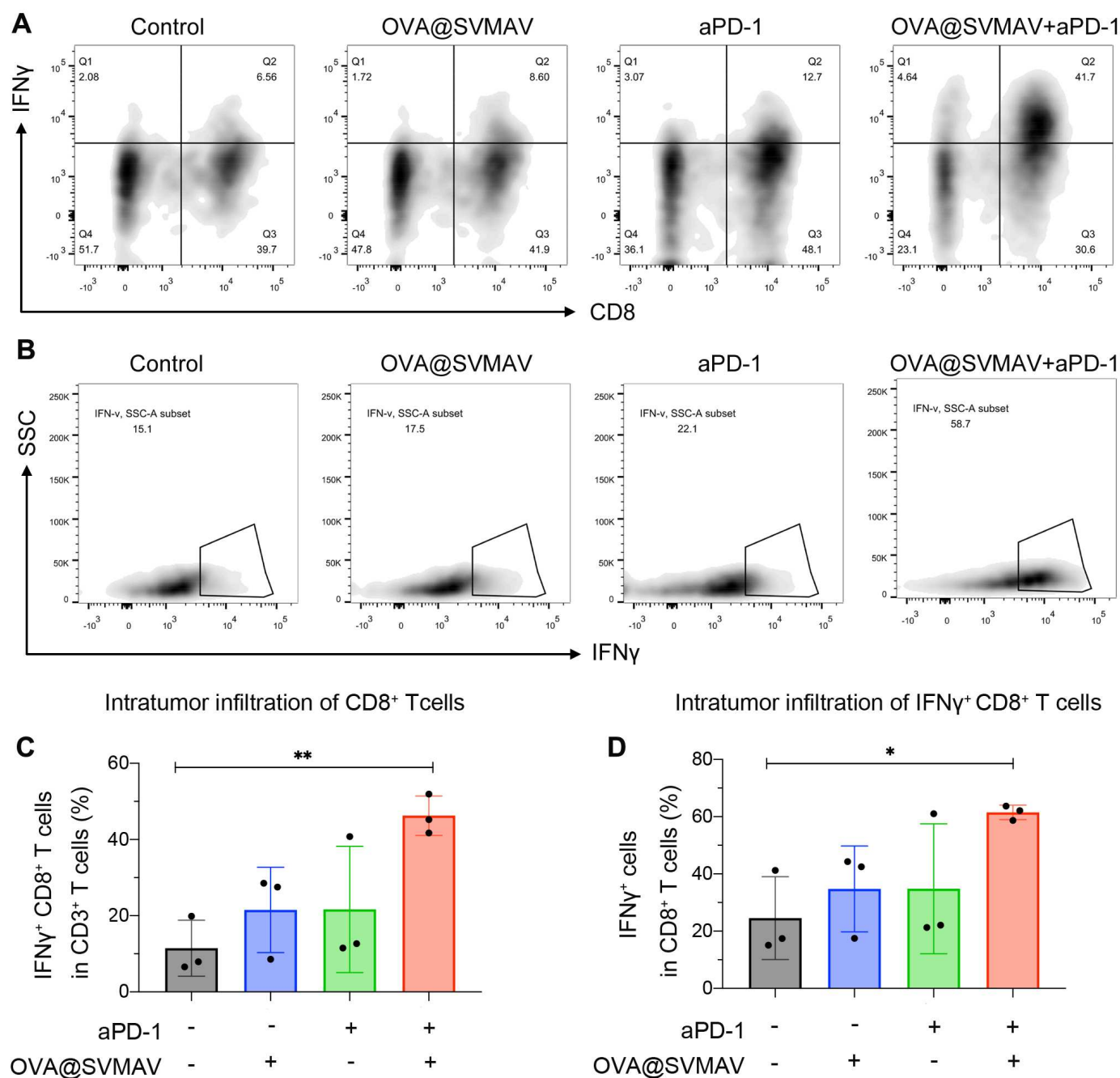

Supplement: Supplementary data [file jitc-2021-003132supp007.pdf]

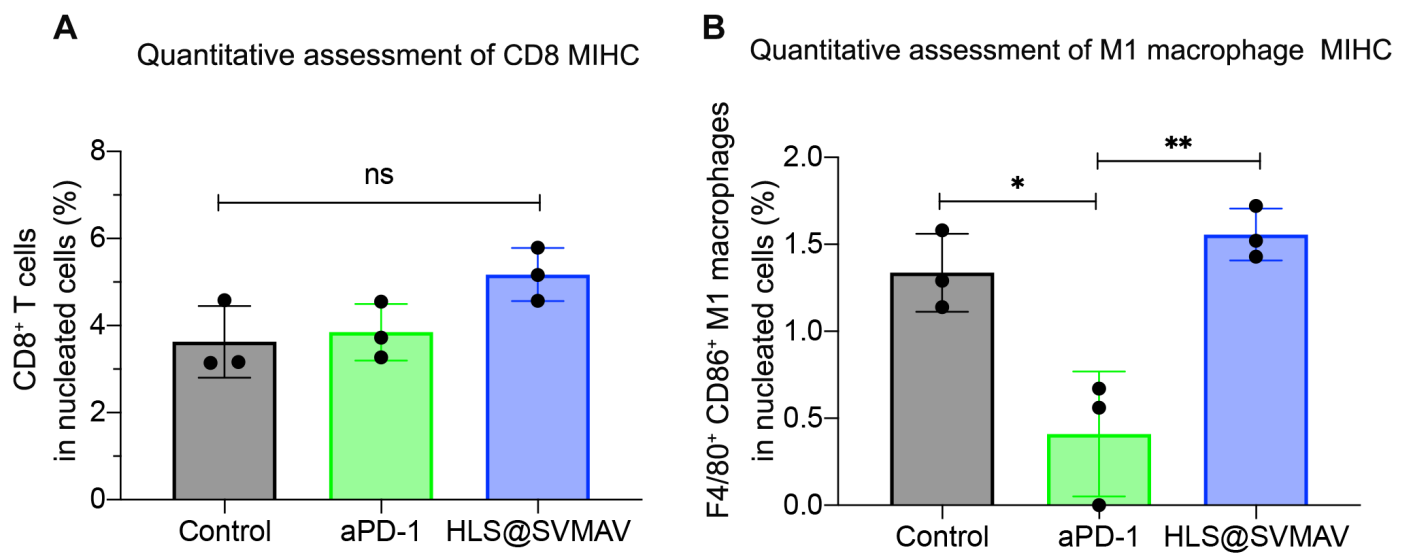

Supplement: Supplementary data [file jitc-2021-003132supp008.pdf]
