# Supplementary material for: Self-assembly nanovaccine containing TLR7/8 agonist and STAT3 inhibitor enhances tumor immunotherapy by augmenting tumor-specific immune response
Source: J Immunother Cancer. 2021 Aug 26;9(8):e003132. doi: 10.1136/jitc-2021-003132 (PMC8404452; doi:10.1136/jitc-2021-003132)
Supplement: Supplementary data [file jitc-2021-003132supp009.pdf]

Self-assembly nanovaccine containing TLR7/8 agonist and STAT3 inhibitor enhances tumor immunotherapy by augmenting tumor-specific immune response

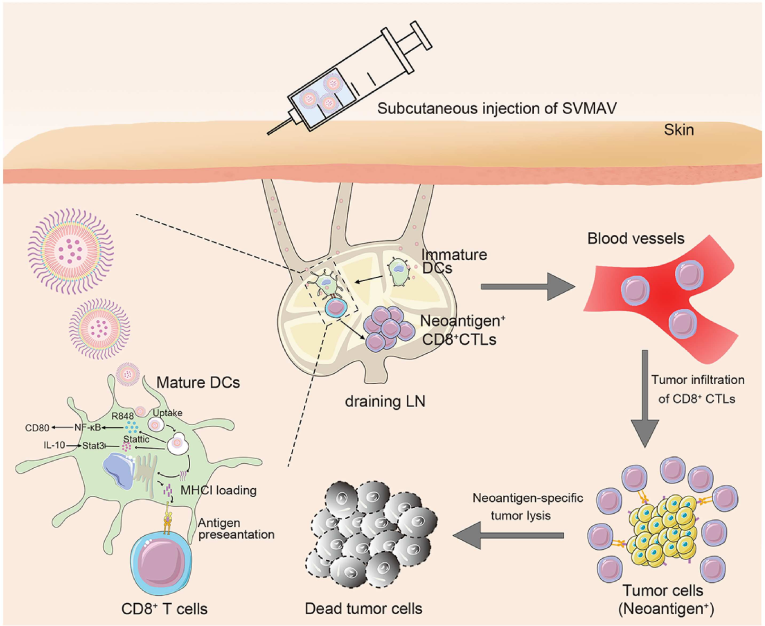

Authors

Lele Zhang, Jiacheng Huang, Xiaona Chen, Caixu Pan, Yong He, Rong Su, Danjing Guo, Shengyong Yin, Shuai Wang, Lin Zhou, Jianxiang Chen, Shusen Zheng, Yiting Qiao

Correspondence

yitingqiao@zju.edu.cn; chenjx@hznu.edu.cn; shusenzheng@zju.edu.cn

In Brief

This work designs a novel cancer nanovaccine named SVMAV, which simultaneously loads antigenic peptides, TLR7/8 agonist R848 and STAT3 inhibitor stattic.

SVMAV possesses abilities of facilitating DC maturation, enhancing antigen cross-presentation and activating T cells.

It also exhibits excellent therapeutic and preventive effect against tumors and metastasis. SVMAV is a potential platform for personalized neoantigen-based cancer vaccines.
